# A ROS‐Responsive and Mucoadhesive Thermosensitive Hydrogel Encapsulated with Mizoribine for Synergistic Dry Eye Therapy

**DOI:** 10.1002/advs.78106

**Published:** 2026-10-06

**Authors:** Danyi Qin, Jiani Li, Panqin Ma, Shinan Wu, Wenying Guan, Yimin Huang, Ke Feng, Mingyan Wei, Zixuan Yang, Shujia Guo, Caihong Huang, Yun‐Long Wu, Yi Han, Zuguo Liu

**Affiliations:** ^1^ Xiamen University affiliated Xiamen Eye Center Fujian Provincial Key Laboratory of Ophthalmology and Visual Science Fujian Engineering and Research Center of Eye Regenerative Medicine; Eye Institute of Xiamen University; School of Medicine Xiamen University Xiamen Fujian China; ^2^ Department of Ophthalmology The First Affiliated Hospital of University of South China Hengyang Medical School University of South China Hengyang Hunan China; ^3^ School of Pharmaceutical Sciences Xiamen University Xiamen Fujian China

**Keywords:** anti‐oxidation, dry eye, mizoribine, ocular surface adhesion, ROS‐responsive, thermosensitive hydrogel

## Abstract

Dry eye disease (DED) is a multifactorial disorder characterized by intricate pathogenesis, necessitating a multi‐target synergistic intervention strategy. Current anti‐inflammatory drugs for DED are limited by low bioavailability, ocular irritation, and inadequate relief. Herein, we propose a multifunctional hydrogel encapsulating Mizoribine, a small‐molecule water‐soluble immunosuppressant. The formulation undergoes in situ gelation on the ocular surface, forms a mucin‐adhered coating, and responds to pathological oxidative stress to enable controlled drug release. In vitro studies demonstrate the formulation effectively mitigates cellular oxidative stress by scavenging ROS and upregulating antioxidant factors. It also protects mitochondrial function, reduces apoptosis, and modulates macrophage polarization. In vivo, topical application of the hydrogel markedly alleviates clinical signs of disease. It also suppresses ocular surface inflammation and reverses conjunctival goblet cell loss. Molecular and cellular analyses indicate the hydrogel exerts its therapeutic effects primarily through antioxidative, anti‐inflammatory, and macrophage‐modulating mechanisms. Importantly, the formulation exhibits an excellent biosafety profile. Our findings highlight that this multifunctional hydrogel serves as a potent, convenient, and safe nanotherapeutic platform. By integrating ROS‐responsive, thermosensitive, and sustained drug‐release properties with antioxidant, anti‐inflammatory, and immunomodulatory functions, this formulation simultaneously targets multiple pathological pathways, offering a promising avenue for clinical translation in treating chronic ocular surface diseases.

## Graphical abstract

The ROS‐responsive in situ thermosensitive MZR@SPPP hydrogel system enables dual‐stage drug loading and sustained ocular delivery. Upon administration, it gelates at ocular surface temperature, enhancing ocular adhesion. By scavenging free radicals, restoring NRF2‐mediated antioxidant defense and mitochondrial homeostasis, and suppressing inflammatory immune responses, this system breaks the dry eye vicious cycle and offers a promising strategy for long‐term management.

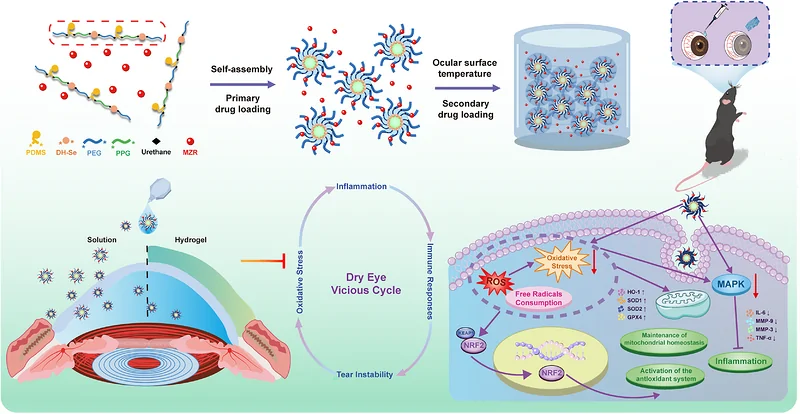

## 1 Introduction

Dry eye disease (DED) is a multifactorial chronic ocular surface disease that can be triggered by any element causing abnormalities in the quality, quantity, or dynamics of tear production. The global prevalence of dry eye is estimated to be 5–50% [1], which is still on a rapid rise [2]. DED patients suffer from ocular discomfort and visual dysfunction [3], and require long‐term management [4]. The prolonged disease course and high recurrence rate significantly affect their quality of life, imposing considerable disease burdens [5]. Tear film homeostasis imbalance, increased tear osmolarity, oxidative stress, ocular surface immune inflammation, and abnormalities in neural regulation are involved in the development of DED [6, 7]. Drugs targeting various pathological mechanisms of DED have been developed. However, significant unmet clinical needs persist due to the inherent limitations of individual agents. Consequently, increasing efforts have been made to develop synergistic therapeutic strategies that offer a more comprehensive approach for DED treatment. Oxidative stress and immune‐mediated inflammation represent two interconnected key mechanisms in the pathogenesis of DED, yet current therapeutic agents often struggle to effectively target both processes simultaneously, underscoring the need for novel pharmaceutical strategies to overcome this challenge. By concurrently alleviating oxidative stress, suppressing inflammatory responses, and restoring immune homeostasis, such strategies hold promise to effectively interrupt the vicious cycle driving disease progression and achieve more robust therapeutic outcomes.

Ophthalmic immunosuppressants like cyclosporine A (CsA) and tacrolimus (FK506), commonly used for moderate to severe DED, face limitations of low bioavailability due to their poor aqueous solubility, typically requiring oily formulations that can cause ocular irritation [8, 9]. In contrast, Mizoribine (MZR) is a low‐molecular‐weight, water‐soluble nucleoside immunosuppressant that suppresses lymphocyte proliferation via a mechanism distinct from that of CsA and FK506 [10, 11, 12, 13]. Our team's previous work has shown MZR to be effective and safe in treating DED [14, 15]. However, small hydrophilic molecules are often rapidly cleared by tears, demanding frequent instillation. Moreover, immunosuppressive agents may exacerbate oxidative stress during their immunomodulatory action. Hopefully, advanced mucoadhesive delivery systems with antioxidative activity provide a promising strategy to prolong its ocular residence and enhance therapeutic efficacy.

In recent years, drug delivery systems have attracted considerable attention in pharmaceutical research, with thermosensitive hydrogels standing out due to their excellent sol‐gel transition properties at physiological temperatures [16]. With the continued development of ophthalmic formulation and drug delivery technologies, stimuli‐responsive drug delivery systems capable of intelligent responses to the pathological microenvironment of ocular diseases, particularly those enabling precise temporal, spatial, and dosage control, have gradually emerged as a major focus of research. These microenvironment‐responsive systems undergo structural or functional changes in response to endogenous (enzymes, metabolites, or oxidative conditions, etc.) or exogenous stimuli (light, ultrasound, or magnetic fields, etc.) to achieve precise and controllable drug delivery. Among these systems, reactive oxygen species (ROS)‐responsive drug delivery platforms offer a unique advantage by enabling site‐specific drug release under oxidative stress while simultaneously scavenging excessive ROS and regulating redox homeostasis. Meanwhile, mucoadhesive drug delivery systems achieve ocular surface adhesion through interactions with epithelial proteins on the ocular surface, thereby prolonging the retention time of drugs in ocular surface tissues, improving bioavailability, and reducing administration frequency. However, current delivery systems still lack sufficient functional integration and are unable to simultaneously fulfill multiple functional requirements, such as ROS responsiveness and ocular surface mucoadhesion.

Based on our existing expertise in nanocarriers for ocular drug delivery, we have developed a ROS‐responsive and mucoadhesive hydrogel eye drop formulation. This copolymer comprises ROS‐responsive, hydrophobic, hydrophilic, and thermosensitive blocks. Upon increasing temperature, the thermally induced phase transition enables secondary drug loading in addition to initial encapsulation, further enhancing the loading capacity for water‐soluble therapeutics. This system aims to extend ocular retention, target multiple pathological mechanisms, and enhance overall therapeutic efficacy, thereby providing novel insights for the development of convenient, efficient, and comprehensive therapeutic approaches for DED and other oxidative stress‐ and inflammation‐related diseases.

## 2 Results and Discussion

### 2.1 Material Characterization

The multiblock copolymer was synthesized from an adhesive block polydimethylsiloxane (PDMS), a selenium (Se)‐based ROS‐responsive block, a thermosensitive poly(propylene glycol) (PPG) block, and a hydrophilic poly(ethylene glycol) (PEG) block, yielding poly(Se‐PDMS‐PEG‐PPG) (SPPP) (Figure 1A).

**FIGURE 1:**
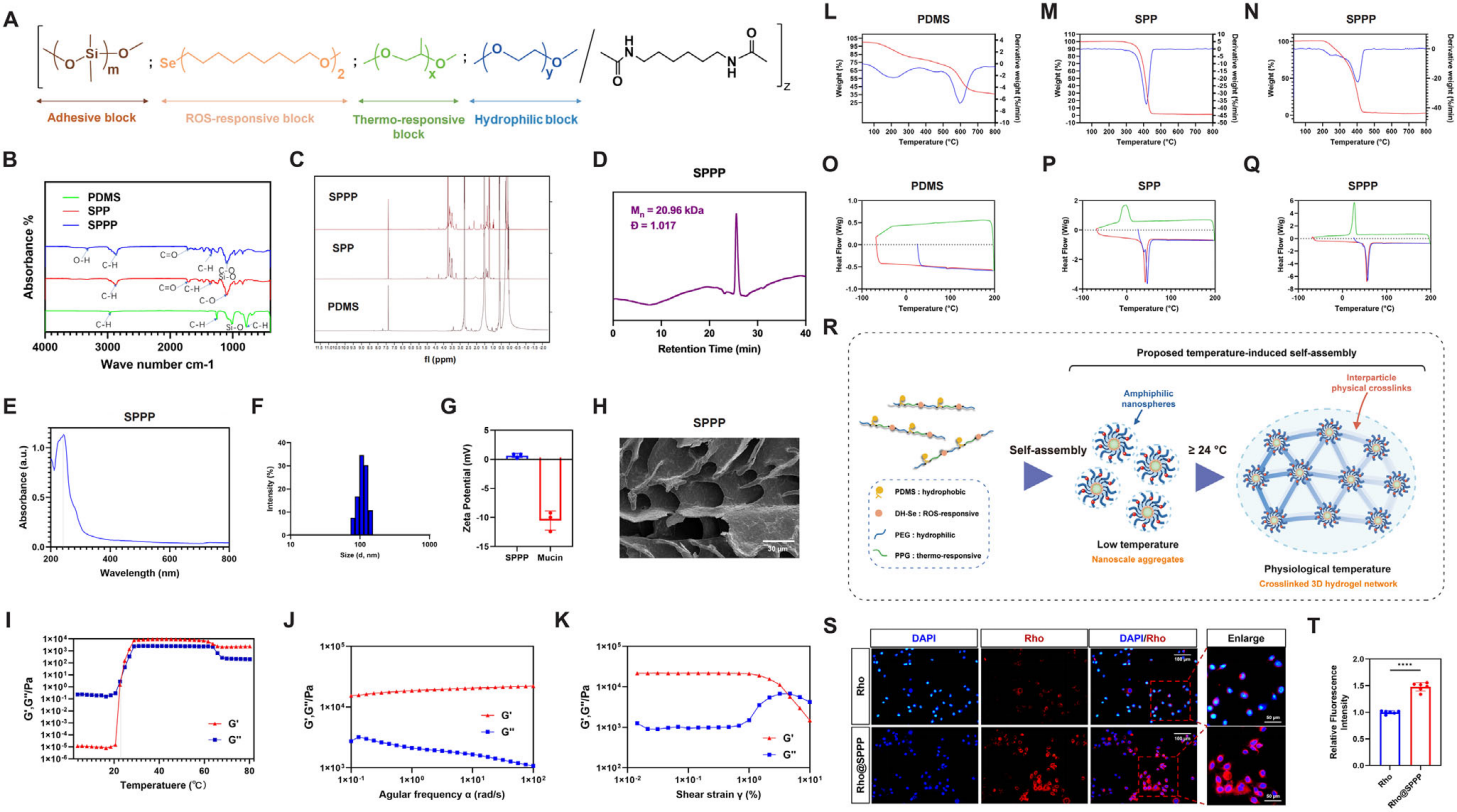
Characterization of SPPP and its cellular uptake. (A) Schematic illustration of the chemical structure of SPPP. (B) FT‐IR spectra of PDMS, SPP, and SPPP. (C) ^1^H NMR spectra of PDMS, SPP, and SPPP in CDCl_3_. (D) GPC spectra of SPPP. (E) Ultraviolet‐visible absorption spectra of SPPP. (F) Size distribution of SPPP. (G) Zeta potentials of SPPP. (H) SEM image of SPPP. TGA curves of PDMS (I), SPP (J), and SPPP (K). DSC cooling and heating curves of PDMS (L), SPP (M), and SPPP (N). Rheological properties of SPPP during temperature sweep (O), frequency sweep (P), and amplitude ramp (Q). (R) An illustrative scheme of the synthesis of the SPPP hydrogel. Fluorescence images of HCE cells (S) and the quantitative analysis of the relative fluorescence intensity (T) (n = 6). Data are presented as mean ± standard deviation (SD); ****p < 0.0001.

Fourier transform infrared (FT‐IR) spectroscopy was conducted to analyze the chemical structure and functional group interactions within the materials. The spectra revealed key vibrational modes and absorption peaks corresponding to functional groups. Polydimethylsiloxane (PDMS) exhibited infrared characteristic peaks of C‐H and Si‐O bonds at 2961, 1257, 1010, and 786 cm^−^^1^, primarily attributed to the siloxane structure (Si‐CH_3_) and skeletal vibrations. Poly(Se‐PEG‐PPG) (SPP) showed infrared characteristic peaks of O‐H, C‐H, C = O, and C‐O bonds at 3332, 2867, 1715, 1342, and 1096 cm^−^^1^, with C‐H and C = O bonds being its typical characteristic peaks. Following this combination, the O‐H characteristic peak in SPPP was observed to shift to 3335 cm^−^^1^. While originating primarily from SPP, this peak showed significantly enhanced intensity, indicating strengthened intermolecular hydrogen bonding within the composite. Likewise, alterations were also noted in the C‐H and C‐O characteristic peak intensities at 2865 and 1342 cm^−^^1^, respectively. The intensity variation at 1342 cm^−^^1^ was ascribed to enhanced hydroxyl and ether bond vibrations following the formation of SPPP (Figure 1B). Enhanced intermolecular hydrogen bonding (O–H and C–H bonds), slight peak shift indicating increased electron cloud density, and preserved characteristic peaks collectively confirmed the successful fabrication of SPPP.

The chemical structures of PDMS, SPP, and SPPP were characterized by ^1^H nuclear magnetic resonance (NMR) (Figure S1) and ^1^ ^3^C NMR (Figure S2). In the ^1^H NMR spectrum, PDMS exhibited characteristic proton signals corresponding to the methyl groups of the siloxane backbone (Figure S1A). The hydroxyl (‐OH) signal originating from SPP disappeared, accompanied by the formation of an O‐Si bond in the SPPP spectrum (Figure 1C). The ^1^ ^3^C NMR spectrum of SPPP revealed diminished Si‐C resonances (∼ 0 ppm) and a concurrent increase in Si‐O‐C peaks (∼ 70 ppm) (Figure S2D). These shifts suggested that methyl groups were substituted by oxygen, transforming Si‐C into Si‐O‐C bonds, indicating successful polymerization.

Gel permeation chromatography (GPC) analysis of SPPP revealed a number‐average molecular weight (Mn) of 20.96 kDa, a weight‐average molecular weight (Mw) of 21.32 kDa, and a narrow dispersity (Đ = 1.02) (Figure 1D), indicating the formation of a relatively uniform multiblock copolymer with a narrow molecular weight distribution.

The ultraviolet‐visible absorption spectrum of SPPP revealed a strong absorption band between 200–300 nm, with a pronounced maximum around 220 nm, which might be attributed to π–π* and n–π* electronic transitions associated with functional groups within the polymer network. Moreover, SPPP exhibited negligible absorbance above 300 nm (Figure 1E), indicating high transparency in the visible light region.

Dynamic light scattering (DLS) results showed that the particle sizes of SPPP and MZR@SPPP ranged from 78.8 to 142 nm (Figure 1F) and 91.3 to 164 nm (Figure S3), respectively. The particle size range fell within the optimal window for nano‐drug delivery systems. The relatively narrow size distribution demonstrated good uniformity. The slight size increase upon drug loading, while still maintaining the nanoscale, indicated successful drug loading without significant aggregation, which was advantageous for drug release and delivery.

Additionally, DLS measurements indicated that SPPP was essentially electrically neutral, with an average zeta potential of ∼0.30 mV (Figure 1G). The near‐neutral surface charge could minimize nonspecific interactions with the negatively charged corneal epithelial cell membrane, thereby eliminating the potential inflammation and cell damage commonly associated with cationic materials [17].

Scanning electron microscope (SEM) images revealed uniformly porous microstructures in both SPPP (Figure 1H) and MZR@SPPP (Figure S4). The pores were well distributed throughout the matrix, indicating successful hydrogel network formation. Importantly, no obvious structural collapse or pore deformation was observed after MZR loading, suggesting that drug incorporation did not significantly alter the internal architecture of the SPPP hydrogel.

Rheological analysis was performed using a temperature‐controlled rheometer to evaluate their thermosensitive and viscoelastic properties. As shown in the temperature sweep curve (Figure 1I), both the storage modulus (G′) and loss modulus (G″) of SPPP increased sharply with increasing temperature, and G′ surpassed G″ at approximately 24°C, indicating a sol–gel transition and confirming the temperature‐induced gelation behavior. Upon further heating, G′ remained consistently higher than G″, demonstrating the formation of a stable gel network. Frequency sweep measurements (Figure 1J) showed that G′ was nearly independent of angular frequency and consistently higher than G″ over the tested range, suggesting a predominantly elastic and well‐developed network structure. In addition, strain sweep analysis (Figure 1K) revealed a clear linear viscoelastic region at low strain, followed by a gradual decrease in G′ at higher strain levels, indicating the onset of network yielding and structural disruption. Collectively, these results confirmed that SPPP exhibited typical thermosensitive gelation behavior and favorable mechanical stability.

The thermal properties of PDMS, SPP, and SPPP were analyzed by thermogravimetric analysis (TGA) (Figure 1L–N) and differential scanning calorimetry (DSC) (Figure 1O–Q). According to the TGA results, PDMS exhibited gradual weight loss starting at 200°C due to thermal decomposition, primarily attributed to the elimination of residual oligomers and pendant methyl groups (Figure 1L). SPP maintained good thermal stability below 300°C with no significant weight loss. The substantial weight loss observed around 400°C was attributed to the decomposition of polyethylene glycol (PEG) and polyvinyl alcohol (PVA) chains. This process generated volatile compounds such as ethers and aldehydes and resulted in rapid mass reduction (Figure 1M). The thermal degradation of SPPP was initiated at around 230°C due to the influence of PDMS. This resulted from the interfacial interactions between PDMS and SPP, which altered the pyrolysis pathway of SPPP and consequently diminished its thermal stability (Figure 1N). The TGA results of SPPP showed that the sample mass remained relatively stable below 200°C, with a loss of less than 5%, indicating good thermal stability in the low to moderate temperature range. This demonstrated that SPPP possessed overall good thermal stability, comparable to that commonly observed in hydrogel biomaterials [18].

According to DSC, PDMS exhibited a low glass transition temperature (Tg) with weak thermal responses, which is characteristic of flexible siloxane chains with high chain mobility (Figure 1O). In contrast, SPP and SPPP displayed more pronounced endothermic and exothermic transitions (Figure 1P,Q). Notably, SPPP exhibited sharper melting and crystallization peaks relative to PDMS and SPP, a result of interfacial interactions that modulated crystallization behavior and enhanced crystal order and homogeneity. Concurrently, the DSC curve of SPPP revealed a single Tg during the heating process, which suggested high miscibility among the multiple polymer components and confirmed the absence of obvious microphase separation (Figure 1Q). These findings indicated that PDMS and SPP were successfully bonded to form the multicomponent copolymer SPPP, rather than a simple physical mixture.

Based on the structure of SPPP and its characterization results, we speculate that SPPP may undergo temperature‐dependent self‐assembly in aqueous solution. At low temperatures, hydration of the PEG and PPG segments helps maintain aqueous dispersibility, while hydrophobic interactions involving the PDMS and PPG segments promote the formation of nanoscale polymer aggregates. Upon increasing temperature, progressive dehydration of the PPG segments enhances interchain hydrophobic interactions, facilitating the association and interconnection of these aggregates into a three‐dimensional hydrogel network (Figure 1R).

Rapid drug internalization by ocular surface cells is highly advantageous for dry eye treatment. To evaluate cellular uptake, human corneal epithelial (HCE) cells were treated with rhodamine (Rho) or Rho@SPPP. Cells exposed to Rho@SPPP exhibited significantly stronger fluorescence within 2 h compared to those treated with free Rho (Figure 1S,T), indicating rapid and efficient internalization by HCE cells. The cellular uptake of nanomaterials involves complex interactions with cell membranes through various mechanisms, such as endocytosis. By facilitating rapid internalization into cells, drugs can evade rapid ocular surface clearance, thereby ensuring prolonged drug retention and sustained therapeutic action within target cells [19]. It also promotes intracellular drug accumulation to achieve a therapeutically effective local concentration. The resulting enhancement in bioavailability may contribute to more efficient intervention in disease‐specific pathways and modulation of cellular functions, leading to improved therapeutic outcomes.

The preliminary storage stability of SPPP and MZR@SPPP formulations was evaluated. Both formulations remained homogeneous without visible precipitation, aggregation, or phase separation during the observation period (Figure S5). DLS analysis further showed minimal changes in particle size and zeta potential, indicating favorable colloidal stability (Figure S6). In addition, high‐performance liquid chromatography (HPLC) analysis demonstrated that the MZR content in MZR@SPPP remained stable after 22 days of refrigerated storage (Figure S7). These results indicate that SPPP and MZR@SPPP formulations exhibit favorable short‐term physical stability under 4°C storage conditions.

### 2.2 Mucoadhesive and Sustained‐Release Properties of MZR@SPPP Hydrogel

SPPP copolymers initially load MZR and self‐assemble into nanoparticles, which subsequently form hydrogels in situ at ocular surface temperature, achieving secondary drug loading (Figure 2A). Upon blinking, the hydrogel conforms to and spreads evenly across the ocular surface (Figure 2B). Macroscopic observations revealed that SPPP formed a transparent hydrogel above 25°C (Figure 2C). Slit‐lamp photography and anterior segment optical coherence tomography (OCT) imaging showed that, compared to Pluronic F127, SPPP hydrogel was uniformly distributed and transparent (Figure 2D). UV–vis transmittance spectra revealed that both Pluronic F127 and SPPP hydrogels exhibited high optical transparency in the visible light range (400–800 nm) (Figure S8).

**FIGURE 2:**
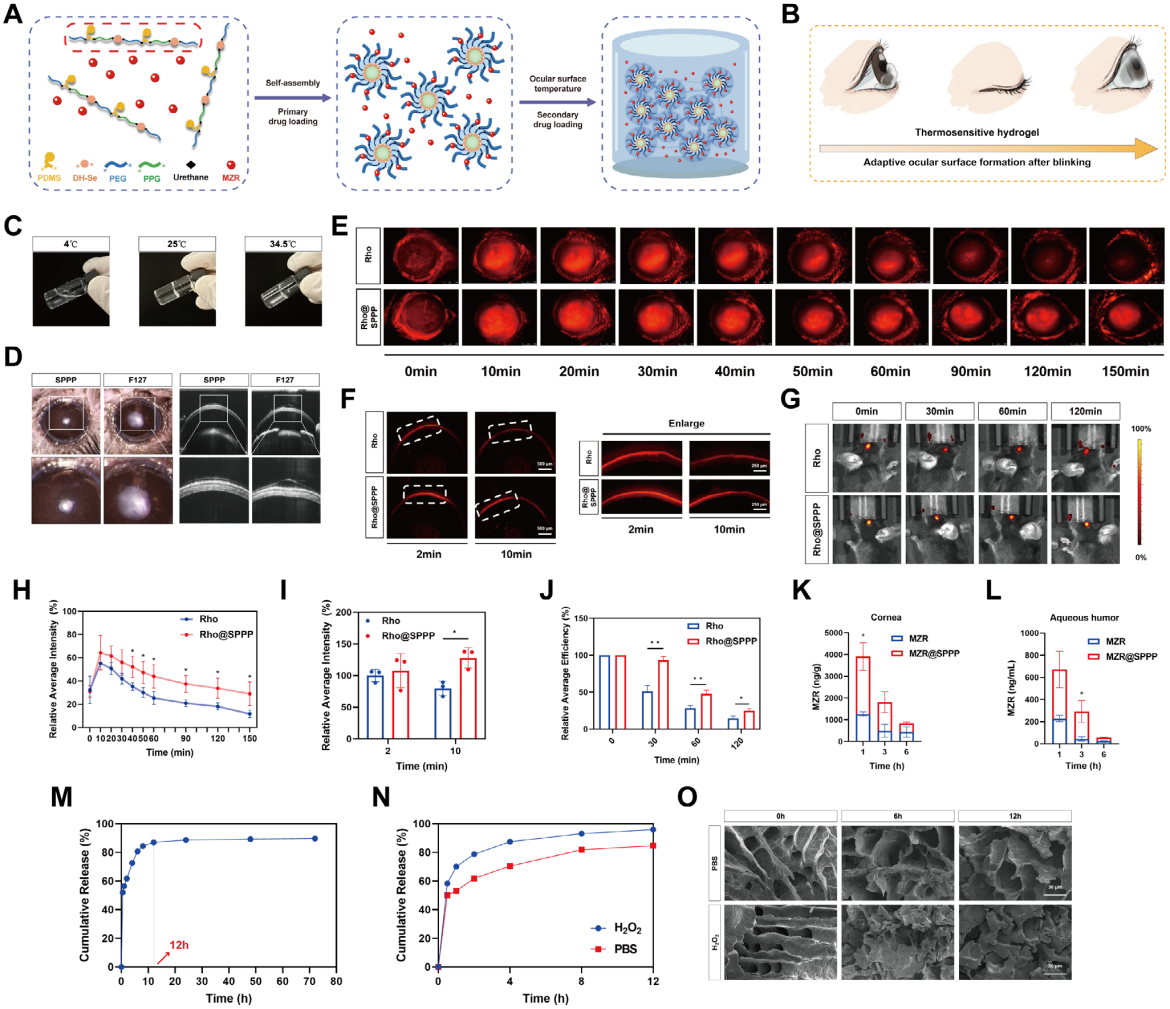
Characterization of the Mucoadhesive and Thermosensitive MZR@SPPP Hydrogel. (A) A schematic diagram illustrating the drug loading process. (B) A diagram showing thermosensitive gelation and adaptive formation on the ocular surface. (C) Macroscopic appearance of SPPP at 4°C, 25°C, and 34.5°C. (D) Slit‐lamp and anterior segment optical coherence tomography images of SPPP and F127. The ocular surface images (E) and quantified average fluorescence intensity (H) (n = 3) at different time points after Rho or Rho@SPPP application. Corneal frozen section images (F) and quantified average fluorescence intensity (I) (n = 3) at different time points after Rho or Rho@SPPP application. In vivo fluorescence images (G) and quantified average fluorescence intensity (J) (n = 3) at different time points after Rho or Rho@SPPP application. Pharmacokinetic profiles of MZR and MZR@SPPP in the cornea (K) and aqueous humor (L) (n = 3). (M) In vitro cumulative drug release profile in PBS. (N) The cumulative drug release profile of MZR@SPPP hydrogel in 500 µM H_2_O_2_. (O) SEM images of SPPP hydrogel incubated in 500 µM H_2_O_2_. Data are presented as mean ± SD; *p < 0.05, **p < 0.01.

The observed difference was likely due to their distinct gelation mechanisms. Pluronic F127 is a triblock copolymer consisting of poly(ethylene oxide) (PEO) and poly(propylene oxide) (PPO) arranged in a PEO–PPO–PEO architecture, in which the hydrophobic PPO core tends to form relatively large hydrophobic domains during thermosensitive gelation [20]. The considerable refractive index mismatch between these domains and water leads to significant light scattering, resulting in localized concentration heterogeneity and diminished transparency. In contrast, SPPP incorporates a PDMS segment characterized by a low T_g_ and high chain flexibility, which facilitates the formation of smaller and more uniform hydrophobic microdomains during gelation. Moreover, the refractive index of PDMS (approximately 1.43) is closer to that of water (about 1.33) [21], achieving better overall refractive index matching and less light scattering. Furthermore, the incorporation of Se‐based groups enables additional intermolecular interactions, facilitating the formation of a more regular and orderly network structure.

Rho was used as a fluorescent indicator to evaluate the ocular adhesion of SPPP. Stereo fluorescence microscopy revealed significant ocular adhesion of SPPP persisting for over 150 min after a single dose (Figure 2E,H). SPPP also markedly enhanced ocular surface permeability, significantly increasing Rho penetration within 10 min post‐application (Figure 2F,I). In vivo imaging further confirmed that SPPP enhanced drug adhesion to the ocular surface (Figure 2G,J). Pharmacokinetic evaluation was performed to characterize the absorption, distribution, and metabolism of MZR in anterior segment tissues, including the cornea and aqueous humor. The results showed that MZR@SPPP achieved higher drug concentrations in these tissues at 1‐ and 3‐h post‐application (Figure 2K,L), indicating higher bioavailability. In vitro drug release studies demonstrated that the release rate of MZR from MZR@SPPP hydrogel increased rapidly to ∼50% within 1 h, reached ∼80% after 6 h, and plateaued after 12 h (Figure 2M), confirming excellent sustained‐release properties. To simulate the pathological microenvironment characterized by local oxidative stress with high ROS levels, a 500 µM hydrogen peroxide (H_2_O_2_) solution was utilized as the release medium [22, 23]. Compared to phosphate buffered saline (PBS), the MZR@SPPP hydrogel exhibited an increased release rate in the 500 µM H_2_O_2_ solution (Figure 2N). To further investigate ROS‐responsive structural changes, SEM analysis was performed under oxidative conditions. Compared with PBS‐treated hydrogels, H_2_O_2_ exposure induced progressive disruption of the porous network, characterized by enlarged pores, reduced network integrity, and partial structural collapse (Figure 2O). These findings provide morphological evidence that ROS stimulation promotes structural remodeling and network loosening of the hydrogel.

Ocular surface adhesion enhancement offers several therapeutic benefits for managing ocular surface diseases. The primary advantage lies in significantly prolonging the residence time of the drug or formulation on the ocular surface, thereby addressing the key pharmacokinetic limitations of conventional ophthalmic products. Traditional eye drops have very brief ocular surface retention, typically lasting less than 5 min, with over 90% of the drug rapidly eliminated through blinking, tear fluid turnover, and nasolacrimal drainage, resulting in an ocular bioavailability generally below 5% [24]. By employing polymers that interact physically or chemically with components of the ocular surface microenvironment, the residence time can be extended to several hours or more. This transitions the drug delivery profile from a brief, pulsed exposure to a sustained, controlled‐release mechanism, ensuring the maintenance of an effective therapeutic concentration over an extended period. These advancements are particularly impactful for chronic ocular conditions such as DED, which typically require long‐term frequent medication. By extending the action duration per dose, the required dosing frequency can be reduced, which helps to improve patient compliance, achieve improved therapeutic outcomes, and ultimately enhance the patient's quality of life [25].

Mucoadhesive drug delivery systems have been considered as a promising strategy for improving drug bioavailability across multiple therapeutic applications [26]. Mucin‐1 (MUC1) is a membrane‐associated mucin extensively expressed on the ocular surface, including corneal and conjunctival epithelial cells, as well as lacrimal gland acinar and ductal cells. It plays an important role in the physiological functions and pathological processes of the ocular surface, primarily serving as a critical component of the mucus layer to maintain tear film stability [27, 28]. Moreover, MUC1 maintains ocular health by enhancing lubrication and providing anti‐inflammatory and immunological defense for the ocular surface. As a widely expressed and membrane‐anchored glycoprotein on ocular cells, MUC1 presents an abundant target for robust adhesion. Pharmaceutical delivery systems may interact with MUC1 to prolong ocular surface residence time, or exert regulatory effects via the activation of intracellular signaling pathways, thereby contributing to enhanced therapeutic efficacy [29]. We observed a strong ocular surface adhesion effect of SPPP, so we further investigated the adhesion mechanism on the ocular surface, exploring whether the interaction between SPPP and MUC1 was involved. We constructed the repeating units of SPPP and MUC1, and performed molecular docking to investigate their interaction patterns and binding affinity (Figure 3A). The results demonstrated that from 0 to 50 ns, both the contact area and the number of hydrogen bonds between SPPP and MUC1 increased rapidly, indicating rapid interaction initiation. The binding reached saturation at approximately 33 ns (Figure 3B–E). Based on these findings, we proposed that SPPP adhered to the ocular surface by interacting with MUC1 expressed on epithelial cells (Figure 3F). To further validate this interaction, we performed surface plasmon resonance (SPR) analysis by immobilizing MUC1 as the ligand on the sensor chip and injecting SPPP or F127 as analytes, in accordance with established methodologies [30, 31]. The results revealed that SPPP exhibited a stronger binding affinity for MUC1 compared to F127, with a kinetic constant (K_D_) of 22 nM for SPPP–MUC1 (Figure 3G), whereas F127 showed much weaker binding, with a K_D_ of 3.64 µM (Figure 3H). These results indicated that the interaction of SPPP and MUC1 might be a key mechanism of ocular surface adhesion, by which SPPP achieves enhanced ocular surface retention. The robust anchoring between SPPP and MUC1 may effectively alleviate the pharmacokinetic limitations of small water‐soluble molecules like MZR, thereby potentially enhancing their bioavailability on the ocular surface.

**FIGURE 3:**
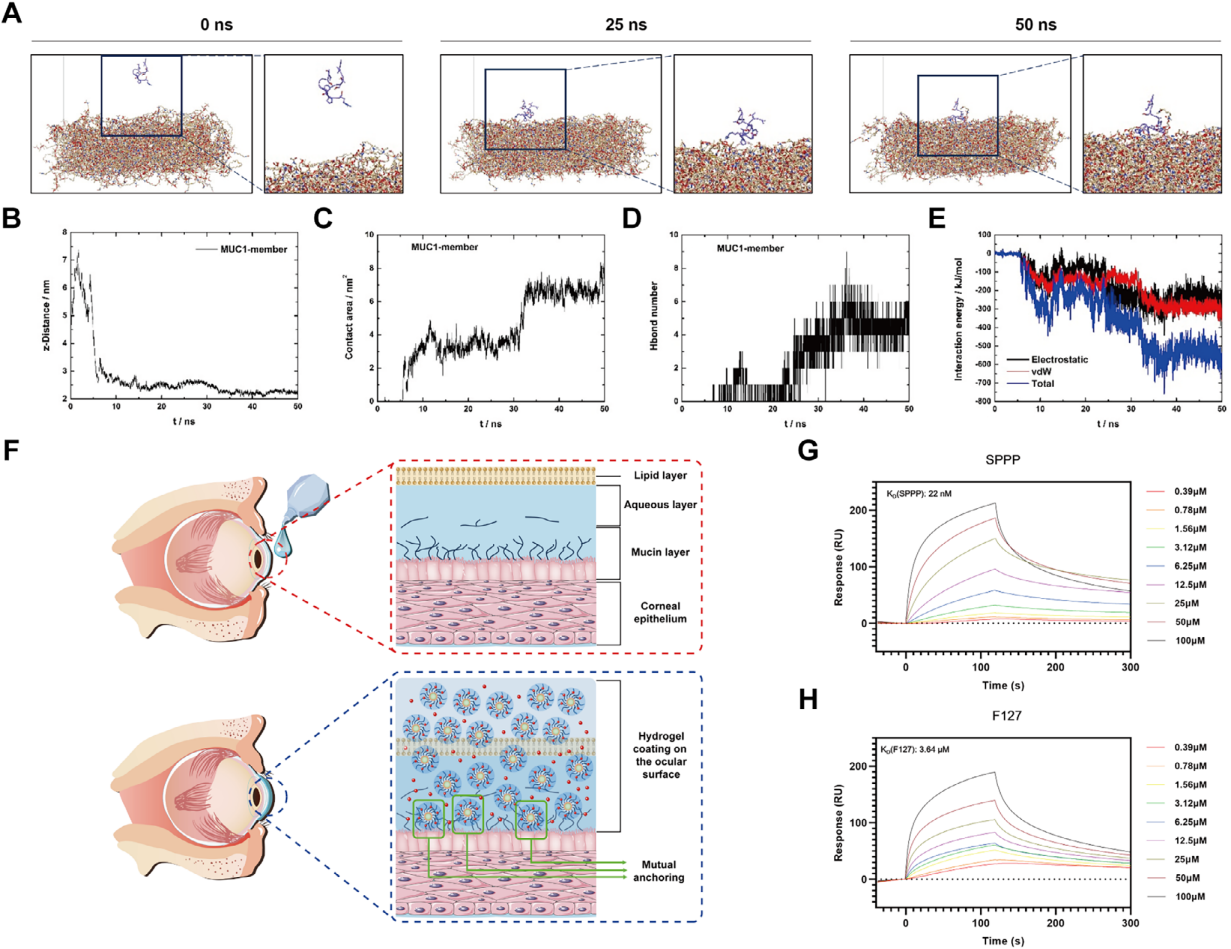
The mechanism underlying the ocular surface adhesion of SPPP. (A) Snapshots from the molecular docking simulation of SPPP and MUC1. The Z‐distance (B), contact area (C), number of hydrogen bonds (D), and interaction energy (E) of SPPP and MUC1, showing their interaction patterns and binding affinity. (F) A schematic diagram illustrating the potential mechanism underlying the ocular surface adhesion of SPPP. SPR evaluation of binding affinity between MUC1 and SPPP (G), or MUC1 and F127 (H).

The interactions between SPPP and MUC1 may originate from the synergistic contribution of multiple reversible non‐covalent interactions. The molecular architecture of SPPP integrates several functional segments that may cooperatively participate in mucoadhesion. Specifically, the ether oxygen groups within PEG/PPG segments and other oxygen‐containing groups in the polymer backbone may serve as hydrogen‐bond acceptors, while hydrogen‐bond donors from the protein and glycan components of MUC1 may facilitate the formation of dynamic hydrogen‐bond networks at the hydrogel–mucin interface. Such multivalent hydrogen bonding has been recognized as an important mechanism underlying polymer–mucin interactions and bioadhesion, as it enables strong yet reversible interfacial attachment under physiological conditions [32]. In addition, the incorporation of PDMS segments into SPPP may further enhance ocular‐surface adhesion through hydrophobic and dispersion interactions. The methyl‐rich siloxane chains of PDMS provide relatively hydrophobic domains that may interact with nonpolar regions of the MUC1 ectodomain, promoting closer interfacial contact between the hydrogel and mucin layer [33]. Compared with F127, which mainly relies on the physical association of PEG/PPG chains, the additional hydrophobic interaction contributed by PDMS may provide complementary adhesive forces and contribute to the stronger SPPP‐MUC1 binding affinity. Furthermore, although the electrostatic attraction between slightly positively charged SPPP and negatively charged mucin components is expected to be relatively weak under physiological ionic conditions, it may provide additional interfacial stabilization. However, our results primarily characterize the overall interaction strength yet cannot quantitatively differentiate the contribution of each individual intermolecular force. Future studies using approaches such as atomic force microscopy‐based single‐molecule force spectroscopy combined with selective functional‐group blocking could further dissect the dynamic adhesion mechanisms.

Regarding the effects of SPPP–MUC1 interactions on the intrinsic hydrogel network structure, we speculate that these interactions primarily enhance local drug exposure by forming a stable yet reversible hydrogel–mucin interface and prolonging hydrogel residence, rather than directly altering drug‐release kinetics within the hydrogel matrix. However, our current data do not directly establish the effects of mucin binding on the network structure or drug‐release behavior. Future studies using mucin‐containing release models and rheological characterization in the presence of mucin are warranted to further clarify these effects.

### 2.3 In Vitro Antioxidant Activity of MZR@SPPP

The human body contains a variety of antioxidants, encompassing macromolecules, small molecules, and enzymes, which act synergistically to scavenge ROS and mitigate oxidative stress. The collective level of these antioxidants reflects the total antioxidant capacity, a parameter of considerable biological significance [34]. In the 2,2′‐azinobis(3‐ethylbenzothiazoline‐6‐sulfonic acid) (ABTS) assay, ABTS is oxidized to a stable green radical cation (ABTS˙^+^) in the presence of an oxidant. By measuring the absorbance of ABTS˙+ at 414 nm or 734 nm, the total antioxidant capacity of the sample can be determined and calculated [35]. As shown in Figure 4A, MZR@SPPP demonstrated a significant antioxidant effect at 50 µg/mL, reaching saturation above 100 µg/mL. In the ferric reducing antioxidant power (FRAP) assay, antioxidants reduce the ferric‐tripyridyltriazine complex to the blue‐colored ferrous form under acidic conditions; the increase in absorbance at 593 nm corresponds to the sample's reducing power [36]. The FRAP results indicated a dose‐dependent increase in the antioxidant capacity of MZR@SPPP (Figure 4B). These results demonstrated that MZR@SPPP exhibited potent antioxidant capacity, as evidenced by the pronounced and correlated responses in both ABTS and FRAP assays [37], supporting its potential to mitigate oxidative stress under biologically relevant conditions.

**FIGURE 4:**
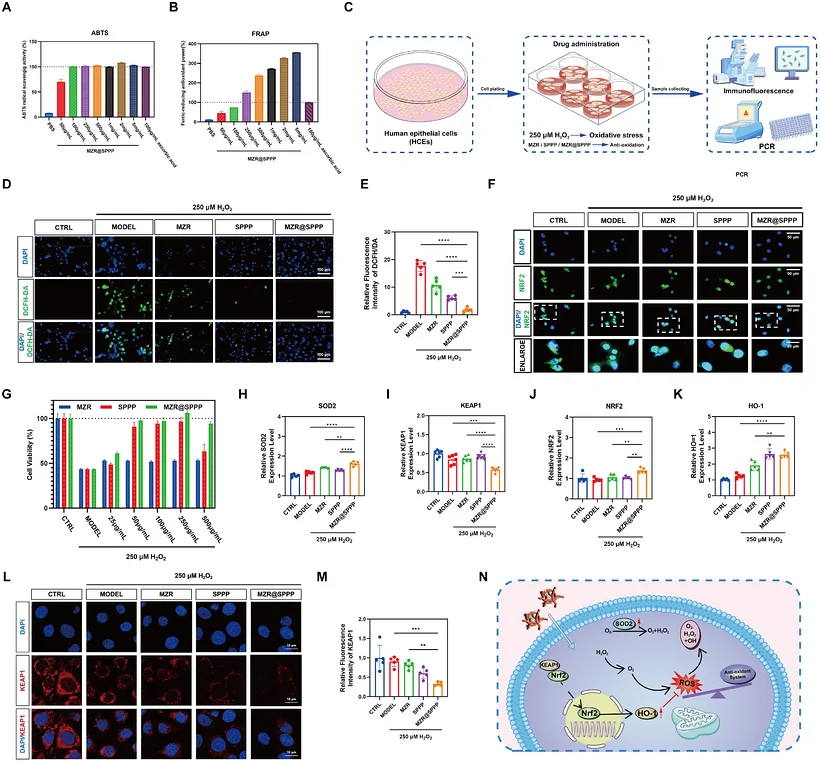
In Vitro Anti‐oxidant Capacity of MZR@SPPP. (A) ABTS radical scavenging activity. (B) Ferric‐reducing antioxidant power. (C) Schematic of the oxidative stress evaluation procedure in HCE cells. Fluorescence images of ROS in HCE cells (D) and quantitative analysis of average fluorescence intensity (E) (n = 5). (F) Immunofluorescence images showing NRF2 localization. (G) Cell viability of HCE cells co‐incubated with H_2_O_2_ and MZR, SPP, or MZR@SPPP. Gene expression levels of SOD2 (H), KEAP1 (I), NRF2 (J), and HO‐1 (K) in HCE cells following different treatments (n = 5). (L) KEAP1 staining images (L) and quantitative analysis of average fluorescence intensity (M) (n = 5). (N) Schematic diagram illustrating the antioxidant mechanism of MZR@SPPP. Data are presented as mean ± SD; **p < 0.01; ***p < 0.001, ****p < 0.0001.

Cellular safety evaluation serves as an initial assessment of potential adverse effects on corneal epithelial cells, allowing early identification of subtle cellular stress, functional impairment, or pro‐inflammatory tendencies [38]. It represents a critical prerequisite for assessing the biocompatibility and translational relevance of biomaterials intended for ocular applications. In vitro experiments confirmed that MZR, SPPP, and MZR@SPPP all exhibited excellent cellular safety in HCE cells at concentrations of up to 500 µg/mL (Figure S9). Live/Dead staining further verified that both SPPP and MZR@SPPP caused little cell death, supporting their favorable biosafety profile (Figure S10). These results suggested that MZR@SPPP exhibited a favorable in vitro biosafety profile, supporting its further evaluation as an ophthalmic functional biomaterial.

The oxidant‐antioxidant imbalance, characterized by deficient superoxide dismutase (SOD) activity and excessive ROS accumulation, may lead to cell death and tissue injury, thereby triggering or aggravating dry eye. To establish an in vitro oxidative stress model, HCE cells were treated with varying concentrations of H_2_O_2_, and cell viability was evaluated at predetermined time intervals. According to the results, exposure to 250 µM H_2_O_2_ for 6 h was chosen as the modeling condition, which resulted in approximately 70% of the normal cell viability in HCE cells (Figure S11), a condition consistent with previous reports [39]. Cell counting kit‐8 (CCK‐8) assay demonstrated that MZR@SPPP markedly restored cell viability at concentrations above 50 µg/mL in this model (Figure 4G). Immunofluorescence staining, quantitative real‐time polymerase chain reaction (qRT‐PCR), and western blotting (WB) were subsequently performed to assess the antioxidant levels (Figure 4C). Intracellular ROS levels, detected by 2′,7′‐dichlorodihydrofluorescein diacetate (DCFH‐DA) assay and fluorescence microscopy, were substantially increased upon H_2_O_2_ exposure compared with the normal control, whereas MZR@SPPP treatment significantly suppressed ROS production (Figure 4D,E). As a key transcription factor regulating antioxidant and detoxifying enzymes, nuclear factor erythroid 2‐related factor 2 (NRF2) activates upon translocation from the cytoplasm to the nucleus. This enables its binding to antioxidant response elements in the promoter regions of target genes, thereby inducing the transcription of a broad array of cytoprotective enzymes involved in redox homeostasis, detoxification, and cellular defense [40]. This critical activation step was confirmed in HCE cells by immunofluorescence staining, which showed the accumulation of NRF2 within the nucleus (Figure 4F). The gene expression levels of anti‐oxidative factors, including SOD2, NRF2, and heme oxygenase‐1 (HO‐1), were significantly upregulated with the treatment of MZR@SPPP, whereas the expression of Kelch‐like ECH‐associated protein 1 (KEAP1), a negative regulator of NRF2 activation, was markedly reduced (Figure 4H–K). Immunofluorescence staining further confirmed the downregulation of KEAP1 expression (Figure 4L,M). These findings suggest that MZR‑loaded hydrogel may enhance the endogenous antioxidant defense system by modulating the KEAP1‐NRF2/HO‐1 signaling axis in HCE cells, thereby alleviating oxidative stress (Figure 4N). Collectively, MZR@SPPP exhibited the strongest antioxidant capacity among all groups, with the antioxidant effect mainly contributed by the SPPP component, while MZR alone showed limited activity.

The KEAP1‐NRF2/HO‐1 pathway is a central antioxidant defense mechanism that maintains cellular redox homeostasis. Under basal conditions, KEAP1 promotes NRF2 degradation and limits NRF2‐dependent antioxidant responses, whereas oxidative stress can disrupt KEAP1‐mediated regulation and facilitate NRF2 activation. In this study, MZR@SPPP treatment reduced KEAP1 expression while enhancing NRF2 and HO‐1 expression, suggesting that the protective effects of MZR@SPPP may involve activation of the KEAP1‐NRF2/HO‐1 antioxidant pathway, thereby alleviating oxidative damage.

### 2.4 In Vitro Mitochondrial Protection of MZR@SPPP

Under the pathological conditions of DED, ocular surface cells are chronically exposed to hyperosmotic and oxidative stress, which profoundly disrupt mitochondrial function. Such mitochondrial impairments promote the activation of apoptotic signaling cascades and amplify local inflammatory responses, ultimately leading to corneal epithelial damage and destabilization of the tear film [41, 42]. To investigate the regulatory effects of MZR@SPPP on mitochondria, we conducted a series of experiments. The terminal deoxynucleotidyl transferase–mediated deoxyuridine triphosphate nick‐end labeling (TUNEL) assay revealed a significant reduction in TUNEL‐positive cells following treatment with MZR@SPPP (Figure 5A). Mitochondrial membrane potential (MMP) is crucial for cellular energy conversion and adenosine triphosphate synthesis; its decline is a recognized hallmark of early apoptosis. Reduced potential from enhanced permeability of the mitochondrial membrane promotes the activation of pro‐apoptotic proteins and facilitates the formation of membrane pores [43, 44]. The fluorescent probe JC‐1 serves as a sensitive indicator of these changes. Under normal conditions, JC‐1 accumulates within the mitochondrial matrix and forms red‐fluorescent J‐aggregates, whereas it exists as green‐fluorescent monomer when membrane potential decreases [45]. JC‐1 staining revealed that H_2_O_2_‐induced oxidative stress significantly reduced MMP, as characterized by a pronounced increase in green fluorescent JC‐1 monomers. In contrast, treatment with MZR@SPPP markedly suppressed this shift, indicating the protective effects in the preservation of mitochondrial integrity and function (Figure 5B,D). Meanwhile, MitoTracker Red CMXRos is a membrane potential–dependent fluorescent probe that selectively accumulates in metabolically active mitochondria of living cells. Its retention within mitochondria is driven by an intact MMP, and a reduction in its fluorescence intensity reflects mitochondrial depolarization and impaired function [46]. Similarly, H_2_O_2_‐induced oxidative stress markedly reduced the population of active mitochondria in HCE cells, and treatment with MZR@SPPP significantly attenuated this loss (Figure 5C,E), indicating a protective effect on mitochondrial integrity under stress conditions. Translocase of outer mitochondrial membrane 20 homolog (TOMM20) is a key receptor protein located on the mitochondrial outer membrane and constitutes an essential component of the translocase of the outer membrane complex. It mediates the initial recognition and docking of nuclear‐encoded mitochondrial preproteins containing N‐terminal targeting sequences and facilitates their translocation across the outer mitochondrial membrane. TOMM20 plays a critical role in maintaining mitochondrial protein homeostasis, biogenesis, and functional integrity. Accordingly, TOMM20 is widely used as a reliable marker of mitochondrial mass and outer membrane integrity in studies investigating mitochondrial structure and function [47]. TOMM20 staining showed that H_2_O_2_‐induced oxidative stress disrupted mitochondrial number, distribution, and morphology, which were restored by MZR@SPPP treatment (Figure 5F). SEM enables detailed observation of mitochondrial ultrastructure, capturing characteristics such as their overall morphology, dimensions, internal cristae organization, and matrix density [48]. Cristae structures, which typically form folds perpendicular to the long axis of the mitochondrion, serve as the primary sites for aerobic respiration and cellular energy production. The number and architectural arrangement of mitochondrial cristae dynamically adapt to the metabolic activity and energy demands of the cell, serving as a structural indicator of mitochondrial functional state [49]. Mitochondrial swelling, cristae architecture disruption, and cytoplasmic vacuolation were observed in HCE cells of the model group, accompanied by the presence of surrounding autophagosomes and lysosomes. Treatment with MZR@SPPP effectively attenuated these ultrastructural abnormalities and preserved mitochondrial integrity (Figure 5G). These results collectively indicated that MZR@SPPP exerted protective effects on mitochondrial structure and function.

**FIGURE 5:**
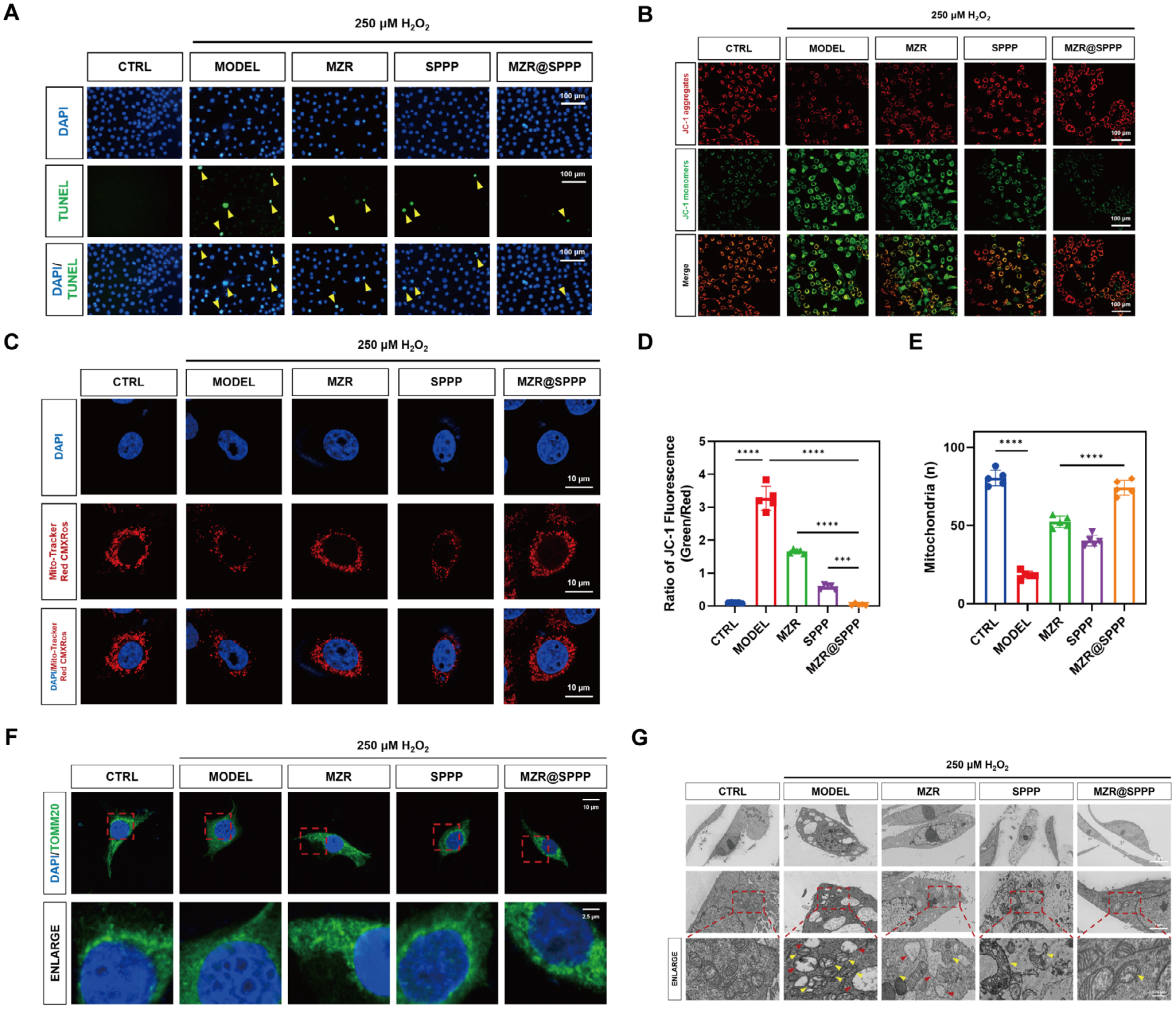
In Vitro Mitochondrial Protective Effect of MZR@SPPP. (A) TUNEL staining images of HCE cells under different treatments. Yellow arrow: TUNEL‐positive cells. JC‐1 staining (red: J‐aggregates; green: monomers) images (B) and quantitative analysis of the JC‐1 red/green fluorescence ratio (D) (n = 5). Mito‐Tracker Red CMXRos staining images (C) and quantitative analysis of the number of mitochondria (E) (n = 5). (F) TOMM20 staining images showing mitochondrial distribution. (G) SEM images showing the intracellular ultrastructure. Yellow arrow: disrupted mitochondria. Red arrow: cytoplasmic vacuoles. Data are presented as mean ± SD, or numbers and percentages (%). ***p < 0.001; ****p < 0.0001.

Mitochondria are recognized as central regulators of cellular homeostasis, which integrate energy metabolism with redox balance, inflammatory signaling, and apoptotic control. Accumulating evidence indicates that mitochondrial dysfunction is a key contributor to the pathogenesis of DED [50], highlighting mitochondrial regulation as a promising therapeutic strategy. Current drug development for DED is increasingly focused on mitochondrial regulation [51, 52, 53]. These drugs mainly function through scavenging ROS and inhibiting the NOD‐like receptor protein 3 inflammasome. In this study, MZR@SPPP exerted a protective effect on corneal epithelial cells by protecting mitochondrial morphology and function, sustaining MMP, and mitigating oxidative stress‐induced damage, ultimately enhancing the survival and functional recovery of ocular surface cells under pathological stress. This mechanism aligns with current advanced strategies, suggesting its potential as a next‐generation therapeutic approach for DED.

To further explore the therapeutic mechanism of MZR@SPPP in the oxidative stress model of HCE cells, transcriptome sequencing was conducted. Compared with the normal control group, the model group exhibited 1187 upregulated and 384 downregulated genes (Figure S12A). In comparison with the model group, the treatment group showed 614 upregulated and 1526 downregulated genes (Figure S12B). By intersecting the differentially expressed genes (DEGs) from Ctrl vs. Model and Model vs. MZR@SPPP comparisons, 585 common DEGs were identified (Figure S13). Gene Ontology (GO) functional annotation revealed the top 20 enriched terms (Figure S14), and Kyoto Encyclopedia of Genes and Genomes (KEGG) pathway analysis highlighted the top 20 enriched terms (Figure S15). Notably, tumor necrosis factor (TNF) and IL‐17 signaling pathways were among the most significantly enriched in the KEGG analysis (Figure S15B). A clustering heatmap illustrating the expression patterns of key DEGs was generated (Figure S16). Significantly, gene set enrichment analysis (GSEA) revealed that MZR@SPPP suppressed the mitogen‐activated protein kinase (MAPK) signaling pathway (Figure S17). qRT‐PCR analysis indicated that MZR@SPPP downregulated the mRNA levels of pro‐inflammatory mediators, including IL‐1β, IL‐6, IL‐8, and TNF‐α (Figure S18). WB confirmed the inhibition of p38 phosphorylation (Figure S19).

DED is recognized as a chronic inflammatory disease in which oxidative stress serves as a key upstream trigger that initiates and sustains inflammatory signaling. Oxidative stress conditions activate stress‐responsive pathways in corneal epithelial cells and induce the expression of pro‐inflammatory cytokines and chemokines, thereby disrupting epithelial homeostasis and compromising tear film stability [54, 55]. Among these, TNF‐ and IL‐17‐associated signaling cascades play pivotal roles in amplifying local inflammation and perpetuating epithelial injury in DED [56, 57]. The p38 MAPK signaling pathway represents a link between oxidative stress and inflammatory gene expression in ocular surface cells. Its activation promotes transcription of multiple inflammatory mediators and is strongly implicated in epithelial damage and disease severity in DED [58]. Therefore, inhibiting MAPK‐driven inflammatory signaling effectively reduces ocular surface damage. In this context, MZR@SPPP facilitates the functional recovery of corneal epithelial cells by promoting the amelioration of the local inflammatory microenvironment.

### 2.5 In Vitro Macrophage Modulation of MZR@SPPP

Macrophages play a pivotal role in shaping the inflammatory microenvironment of the ocular surface and are actively involved in the initiation and amplification of inflammatory responses in DED. Macrophages can respond to disease‐associated signals and adopt distinct functional phenotypes to profoundly shape the course of disease progression [59]. To investigate the immune regulatory effects of MZR@SPPP, we induced RAW264.7 into M1 macrophages using lipopolysaccharide (LPS) and interferon‐gamma (IFN‐γ), and into M2 macrophages using IL‐4 and IL‐13, and then evaluated the capacity of MZR@SPPP to suppress M1 and promote M2 polarization (Figure 6A). Immunofluorescence staining showed increased expression of cluster of differentiation 86 (CD86) and inducible nitric oxide synthase (iNOS) in the model group, which was attenuated by MZR@SPPP treatment (Figure 6B). Meanwhile, MZR@SPPP treatment markedly enhanced the expression of M2 markers cluster of differentiation 206 (CD206) and arginase‐1 (Arg‐1) (Figure 6C). qRT‐PCR analysis substantiated that MZR@SPPP effectively reduced the mRNA levels of key M1 markers, including IL‐6 (Figure 6D), iNOS (Figure 6E), and C‐C motif chemokine ligand 2 (CCL2) (Figure 6F), and significantly increased the gene expression levels of the M2 markers IL‐10 (Figure 6G) and chitinase‐like 3 (CHIL3) (Figure 6H). Moreover, flow cytometric measurement of CD86 and CD206 fluorescence intensity provided additional confirmation of the inhibitory effect of MZR@SPPP on M1 macrophages (Figure 6I,J) and the promoting effect on M2 polarization (Figure 6K,L). Enzyme‐linked immunosorbent assay (ELISA) results showed that MZR@SPPP treatment significantly reduced the secretion of IL‐1β and TNF‐α while enhancing IL‐10 production. Compared with MZR and SPPP, MZR@SPPP exhibited superior regulatory effects (Figure 6M–O).

**FIGURE 6:**
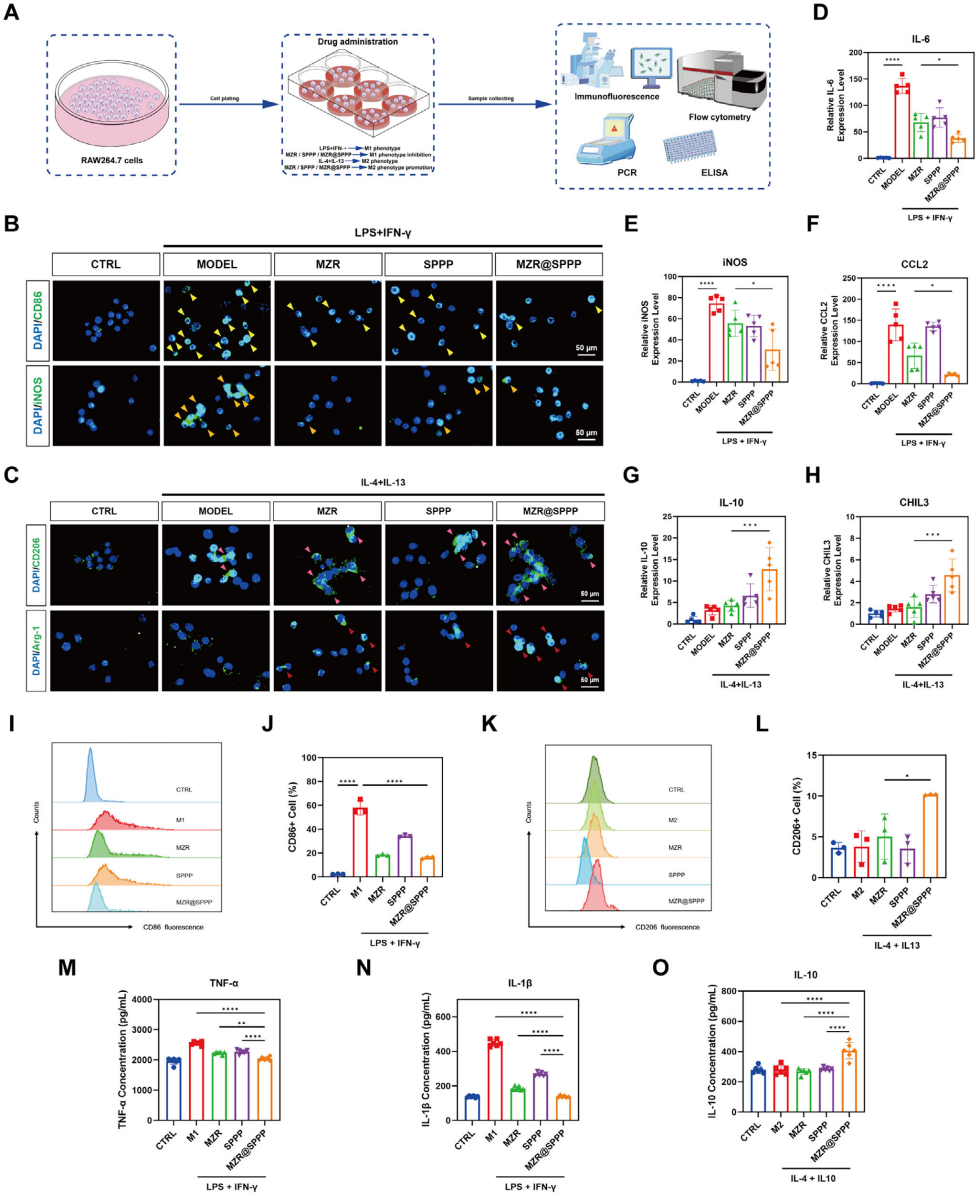
In Vitro Modulation of Macrophage Polarization Shift. (A) Schematic of RAW264.7 polarization evaluation procedure. (B) Immunofluorescence images showing M1 markers CD86 and iNOS. Yellow arrow: CD86‐positive cells. Orange arrow: iNOS‐positive cells. (C) Immunofluorescence images showing M2 markers CD206 and Arg‐1. Pink arrow: CD206‐positive cells. Red arrow: Arg‐1‐positive cells. Relative gene expression levels of IL‐6 (D), iNOS (E), CCL2 (F), IL‐10 (G), and CHIL3 (H) by qRT‐PCR. (I) Flow cytometry analysis of CD86+ RAW264.7 cells. (J) Quantitative analysis of the proportions of CD86+ RAW264.7 cells. (K) Flow cytometry analysis of CD206+ RAW264.7 cells. (L) Quantitative analysis of the proportions of CD206+ RAW264.7 cells. (M) ELISA quantification of TNF‐α concentrations. (N) ELISA quantification of IL‐1β concentrations. (O) ELISA quantification of IL‐10 concentrations. Data are presented as mean ± SD. *p < 0.05; **p < 0.01; ***p < 0.001; ****p < 0.0001.

Although RAW264.7 cells are widely used for macrophage activation studies, this immortalized cell line may not fully recapitulate the characteristics of primary ocular surface macrophages. Moreover, monoculture systems cannot reproduce the complex interactions within the ocular immune microenvironment. Therefore, the immunomodulatory effects observed in RAW264.7 cells should be interpreted as an initial evaluation of the direct effects of MZR@SPPP. Future studies using primary macrophages, human macrophage models, and multicellular coculture systems are warranted to further validate these effects.

### 2.6 In Vivo Evaluation of MZR@SPPP Thermosensitive Hydrogel Eye Drops in DED

The tissue compatibility of hydrogel‐based drug delivery systems is critical for their applications on the ocular surface, as they must be well‐tolerated by delicate ocular tissues without inducing adverse reactions [60]. To assess potential cytotoxicity and irritancy, we performed a series of in vivo evaluations (Figure S20). Following a 7‐day topical administration, slit‐lamp photography revealed that neither SPPP nor MZR@SPPP caused significant damage to the corneal epithelium (Figure S21). Anterior segment OCT (Figure S22) and in vivo confocal microscopy (Figure S23) revealed no obvious structural alterations in the cornea, including epithelium, nerves, stroma, or endothelium. In the posterior segment, no notable structural alterations were observed in the fundus revealed by fundus photography or OCT (Figure S24), and visual function assessed by electroretinography demonstrated no significant adverse effects by SPPP or MZR@SPPP (Figure S25).

To further verify the long‑term ocular and systemic safety of SPPP and MZR@SPPP, we extended our safety evaluation to a 1‑month observation period. No appreciable pathological lesions were identified throughout this extended experiment. Specifically, no apparent injury to the corneal epithelium (Figure S26) or detectable structural changes in the anterior‐segment tissues (Figure S27) were observed, and no prominent changes were found across all corneal layers (Figure S28). Similarly, no abnormalities were found in the morphology (Figure S29) or functional status (Figure S30) of the posterior segment. Hematoxylin and eosin (H&E) staining of ocular tissues demonstrated the absence of overt pathological changes in the cornea, conjunctiva, and retina (Figure S31). Major systemic organs, including the heart, liver, spleen, lung, and kidney, exhibited no obvious histopathological alterations (Figure S32). In addition, no significant changes were observed in complete blood count (Figure S33) or serum biochemical parameters (Figure S34).

To evaluate the therapeutic effects of MZR@SPPP on dry eye in vivo, we established a mouse model of dry eye by subjecting animals to 7‐day sustained subcutaneous injection of scopolamine combined with exposure to a controlled low‐humidity and high‐airflow environment. As an anticholinergic agent, scopolamine significantly reduces tear secretion by inhibiting parasympathetic nerve excitation and competitively inhibiting muscarinic M receptors in the lacrimal glands. Meanwhile, the low‐humidity environment generated by controlled environment chambers accelerates tear film hyper‐evaporation, collectively inducing desiccating stress (DS) (Figure 7A). The method is widely employed for dry eye modeling, as it primarily induces dry eye symptoms by damaging corneal epithelial cells and accelerating tear film breakdown [61]. During the modeling process, mice from the respective groups received twice‐daily topical administration of different eye drops (PBS, MZR, SPPP, MZR@SPPP, and CsA) for 7 days. By day 7, mice in the model group exhibited typical clinical signs of dry eye, including corneal epithelial defects and reduced tear secretion, indicating the successful establishment of the dry eye model. Corneas from mice in the model and vehicle control groups exhibited intense fluorescence, with staining scores >7, indicating pronounced dry eye symptoms [62]. Monotherapies, including MZR, SPPP, MZR@SPPP, and CsA, significantly reduced corneal fluorescence in varying degrees. The MZR@SPPP treatment group demonstrated significant therapeutic outcomes, as evidenced by markedly reduced corneal staining scores, increased tear secretion, and prolonged tear break‐up time (TBUT) (Figure 7B–E). Notably, MZR@SPPP exhibited comparable efficacy to commercial CsA in promoting corneal epithelial repair (Figure 7C), while surpassing CsA in enhancing tear production (Figure 7D). The dry eye model induced solely by benzalkonium chloride (BAC) instillation also demonstrated significant therapeutic efficacy with MZR@SPPP treatment, as evidenced by markedly reduced corneal epithelial defects, enhanced tear secretion, and entended TBUT (Figure S35). H&E staining revealed that the corneal epithelium in the model group exhibited structural disruption and desquamation, with its thickness reduced by approximately half compared to the normal control group. Concurrently, the corneal stroma appeared thickened and looser, containing an excessive number of vacuolar structures, findings that align with previously reported pathological features in similar models [63]. The conjunctival epithelium in the model group also exhibited a significant reduction in thickness. Treatment with MZR@SPPP markedly restored the morphological and structural integrity of both the corneal and conjunctival tissues (Figure 7F). Periodic acid‐Schiff (PAS) staining demonstrated a significant reduction in conjunctival goblet cell density in the model group. In contrast, treatment with MZR@SPPP greatly attenuated goblet cell loss induced by DS (Figure 7G,H). Immunofluorescence staining of keratin 10 (KRT10) and keratin 12 (KRT12) further evaluated corneal epithelial phenotype changes. Compared with the normal control, the model group showed increased KRT10 and decreased KRT12 expression, indicating a shift toward a squamous‐like epithelial phenotype. MZR@SPPP treatment markedly reduced KRT10 expression and restored KRT12 expression (Figure 7I–L), suggesting that MZR@SPPP alleviated dry eye‐associated epithelial phenotypic alterations. SEM revealed distinct morphological differences among the groups. The corneal epithelial surface in the normal control group was continuous and well‐organized, displaying tightly interlocked microplicae that indicated intact barrier function and cellular integrity. In contrast, the model group exhibited significant surface disruption, characterized by epithelial desquamation, a reduced density of microplicae, and widened intercellular spaces. Treatment with MZR@SPPP markedly improved the corneal morphology, showing a restored cellular arrangement, reappearance of microvilli and microplicae, and improved surface regularities (Figure S36). Collectively, both free MZR and the blank SPPP hydrogel alone provided partial relief of dry eye signs, whereas MZR@SPPP yielded the most pronounced therapeutic outcomes. This superior efficacy highlights a potent synergistic effect, jointly creating an optimal microenvironment for corneal repair.

**FIGURE 7:**
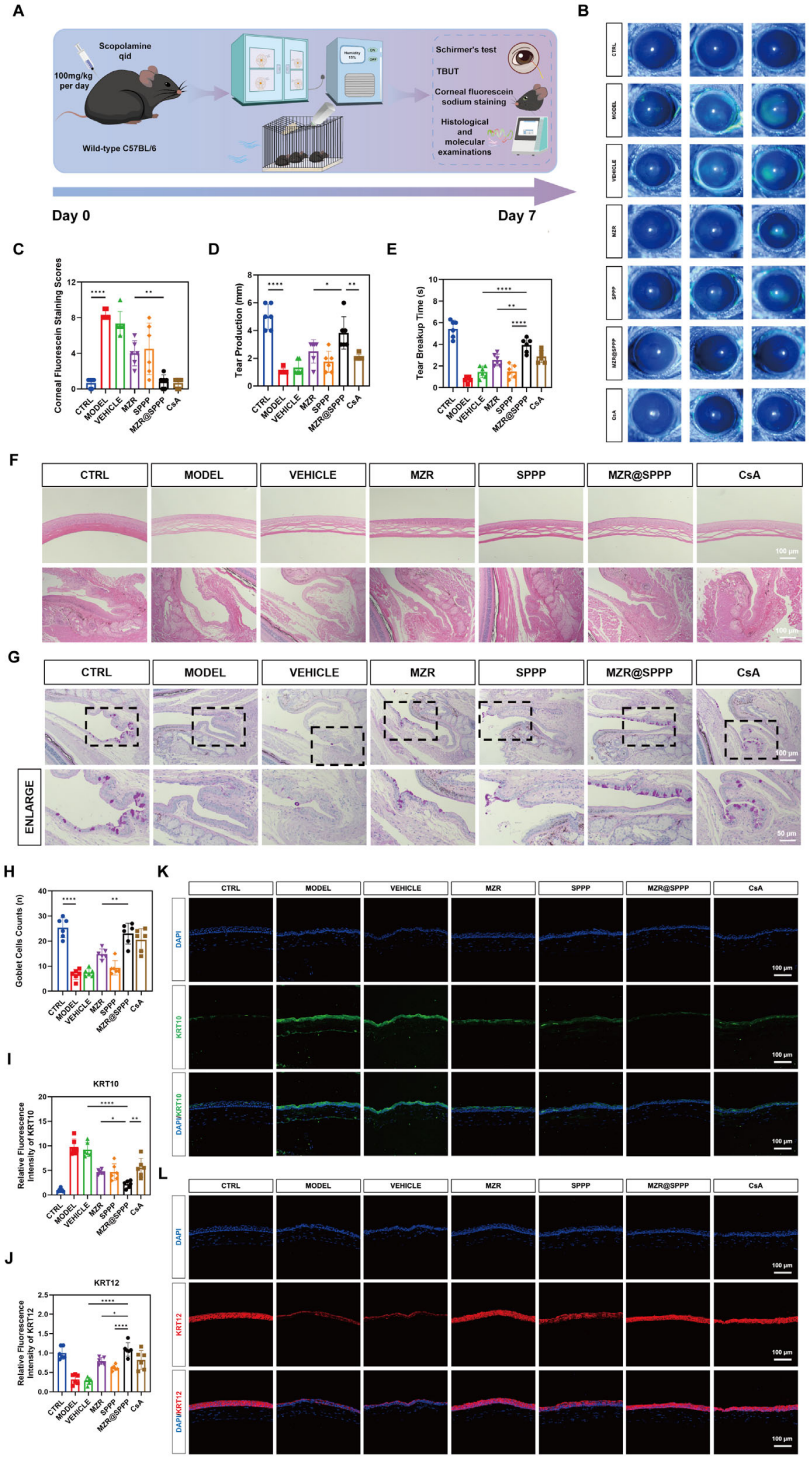
In Vivo Evaluation of MZR@SPPP Hydrogel Eye Drops in DED. (A) A schematic diagram illustrating the establishment of the dry eye mouse model. (B) Corneal fluorescein staining images. (C) Quantitative analysis of corneal fluorescein staining scores (n = 6). (D) Quantitative analysis of tear production (n = 6). (E) Quantitative analysis of TBUT (n = 6). (F) H&E staining images of the ocular surface. (G) PAS staining images of conjunctival sections. (H) Quantitative analysis of conjunctival goblet cells (n = 6). Statistical analysis of relative fluorescence intensity of KRT10 (I) and KRT12 (J) (n = 6). Immunofluorescence images of KRT10 (K) and KRT12 (L) in corneas. Data are presented as mean ± SD or numbers and percentages (%); *p < 0.05; **p < 0.01; ***p < 0.001; ****p < 0.0001.

In our study, MZR@SPPP hydrogel eye drops demonstrated promising potential for treating DED. Compared to MZR alone, MZR@SPPP exhibited significantly enhanced therapeutic efficacy, primarily attributed to improved drug bioavailability achieved through formulation optimization and synergistic antioxidant activity inherent to SPPP. Notably, MZR@SPPP showed comparable effectiveness to CsA in promoting corneal epithelial repair, while exhibiting superior performance in improving tear secretion. These findings, together with previous research from our group [15], collectively validate the clinical translation potential of MZR and its formulations. When compared to other hydrogel‐based dry eye treatments reported in recent years, MZR@SPPP exhibits comparable or superior overall efficacy [31, 64, 65], yet distinguishes itself through the unique properties of both the carrier and the encapsulated drug. In particular, MZR, as a small‐molecule water‐soluble immunosuppressant, offers distinctive advantages in its mechanism of action and biocompatibility relative to conventional macromolecular or lipophilic agents. This uniqueness constitutes the core innovation and competitive strength of our drug delivery system.

However, several limitations should be considered regarding their clinical translation. The scopolamine combined with DS mouse model is one of the most widely used experimental DED models [66, 67], and provides a reproducible platform for evaluating therapeutic efficacy and investigating disease‐related mechanisms [68, 69]. However, human DED is a complex and heterogeneous chronic disease involving diverse etiologies, whereas the scopolamine and DS model primarily reflects aqueous deficiency and environmentally induced ocular surface stress. The relatively short induction period, together with species differences, may limit its ability to fully recapitulate the persistent and recurrent nature of human DED. Therefore, the therapeutic and safety effects observed in this study should be regarded as preclinical proof‐of‐concept evidence. Future studies using diverse dry eye models, large‐animal models, and ultimately clinical trials in humans are warranted to further validate the therapeutic efficacy of MZR@SPPP.

Beyond this, the degradation characteristics and long‐term biosafety of the polymeric carrier are also critical considerations for clinical translation. The selenide bonds within the polymer backbone are susceptible to ROS‐mediated oxidation, resulting in increased polymer polarity, weakened hydrophobic interactions, and subsequent hydrogel network loosening and structural disassembly. Different components of SPPP may undergo distinct degradation pathways. The selenium‐containing segments may generate oxidized organ selenium species, whereas the PEG and PPG polyether segments may undergo oxidative chain scission to produce lower‐molecular‐weight polyether fragments. The PDMS segments may experience gradual oxidation of siloxane bonds, resulting in shorter siloxane‐containing fragments. Subsequently, continuous clearance from the ocular surface via tear turnover, blinking, and nasolacrimal drainage, can facilitate the elimination of these residual fragments and degradation intermediates, thereby mitigating the risk of prolonged systemic accumulation. Meanwhile, several selenium‐containing polymers have previously been explored as drug delivery platforms and have demonstrated favorable cytocompatibility and in vivo tolerability. Consistent with these previous reports, our safety evaluations further demonstrated the favorable ocular and systemic biocompatibility. Nevertheless, we acknowledge that direct quantitative assessment of SPPP degradation kinetics, selenium‐containing degradation products, and their long‐term metabolic fate remains necessary. Future studies incorporating selenium quantification using inductively coupled plasma mass spectrometry, and long‐term pharmacokinetic and biodistribution evaluations will provide further insights into the potential for clinical translation. Moreover, previous studies have demonstrated favorable cytocompatibility and in vivo tolerability of selenium‐containing polymers [23, 70], which aligns well with our safety evaluation results. Nevertheless, further quantitative investigations of degradation kinetics, selenium‐containing degradation products, and their long‐term metabolic fate are warranted to fully elucidate the translational potential of this platform.

### 2.7 Mechanism Investigation of MZR@SPPP in Alleviating DED

To elucidate the therapeutic mechanisms of MZR@SPPP in DED, we performed transcriptome sequencing on corneal tissues from the normal control (Ctrl), dry eye model (Model), and MZR@SPPP‐treated group (MZR@SPPP). RNA sequencing (RNA‐seq) analysis identified 2487 upregulated and 2447 downregulated genes between Ctrl and Model groups (Figure 8A). And MZR@SPPP treatment resulted in 2850 DEGs, with 1475 genes upregulated and 1375 downregulated (Figure 8B). The intersection of DEGs from the Ctrl vs. Model and Model vs. MZR@SPPP comparisons revealed 398 genes (Figure 8C). GO enrichment analysis identified the top 20 significantly enriched terms (Figure 8D,E), while KEGG pathway analysis highlighted the top 20 enriched pathways (Figure 8F,G). Notably, nuclear factor kappa‐B (NF‐κB), IL‐17, TNF, and MAPK signaling pathways, which are closely linked to the inflammatory processes in DED, were among the most significantly enriched pathways (Figure 8G). Additionally, a clustering heatmap was generated to visualize the expression patterns of the key DEGs (Figure 8H).

**FIGURE 8:**
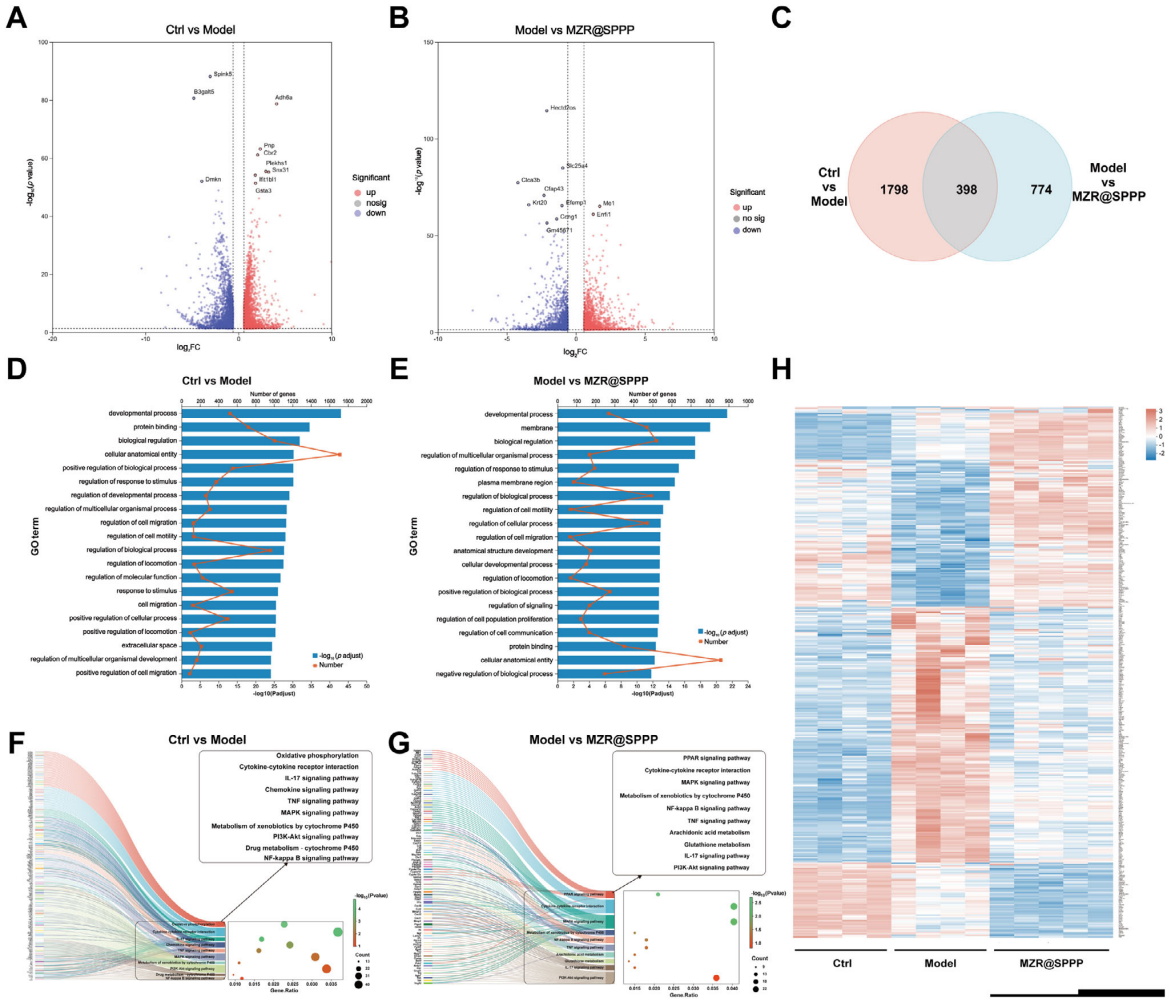
Overview of Transcriptome Sequencing. (A) A volcano plot visualizing the DEGs between Ctrl and Model groups. (B) A volcano plot visualizing the DEGs between Model and MZR@SPPP groups. (C) A Venn diagram showing the overlap of common DEGs between Ctrl vs. Model and Model vs. MZR@SPPP. (D) GO enrichment analysis of DEGs between Ctrl and Model groups. (E) GO enrichment analysis on the DEGs between Model and MZR@SPPP groups. (F) KEGG pathway enrichment analysis of DEGs between Ctrl and Model groups. (G) KEGG pathway enrichment analysis on the DEGs between Model and MZR@SPPP groups. (H) A heatmap displaying the expression levels of genes across different groups.

The ocular surface is directly exposed to the external environment, making it susceptible to oxidative stress from various external insults such as ultraviolet radiation, blue light, and air pollutants [71]. Oxidative damage can lead to cell death and ocular surface impairment, which contributes to the development and progression of DED [72]. Targeting oxidative stress represents a key therapeutic strategy for DED, as it disrupts the pathogenic cycle of oxidative stress, inflammation, apoptosis, and neurodegeneration. RNA‐seq revealed that MZR@SPPP effectively downregulated the expression of oxidative stress‐related genes and suppressed associated pathways (Figure 9A,B). Furthermore, qRT‐PCR analysis confirmed that MZR@SPPP upregulated the mRNA levels of key antioxidant genes, including NRF2, HO‐1, SOD1, SOD2, and glutathione peroxidase 4 (GPX4), in corneal tissues (Figure 9C–G). Consistently, immunofluorescence staining revealed a significant elevation of NRF2 expression in the corneal epithelium following MZR@SPPP treatment (Figure 9H,I).

**FIGURE 9:**
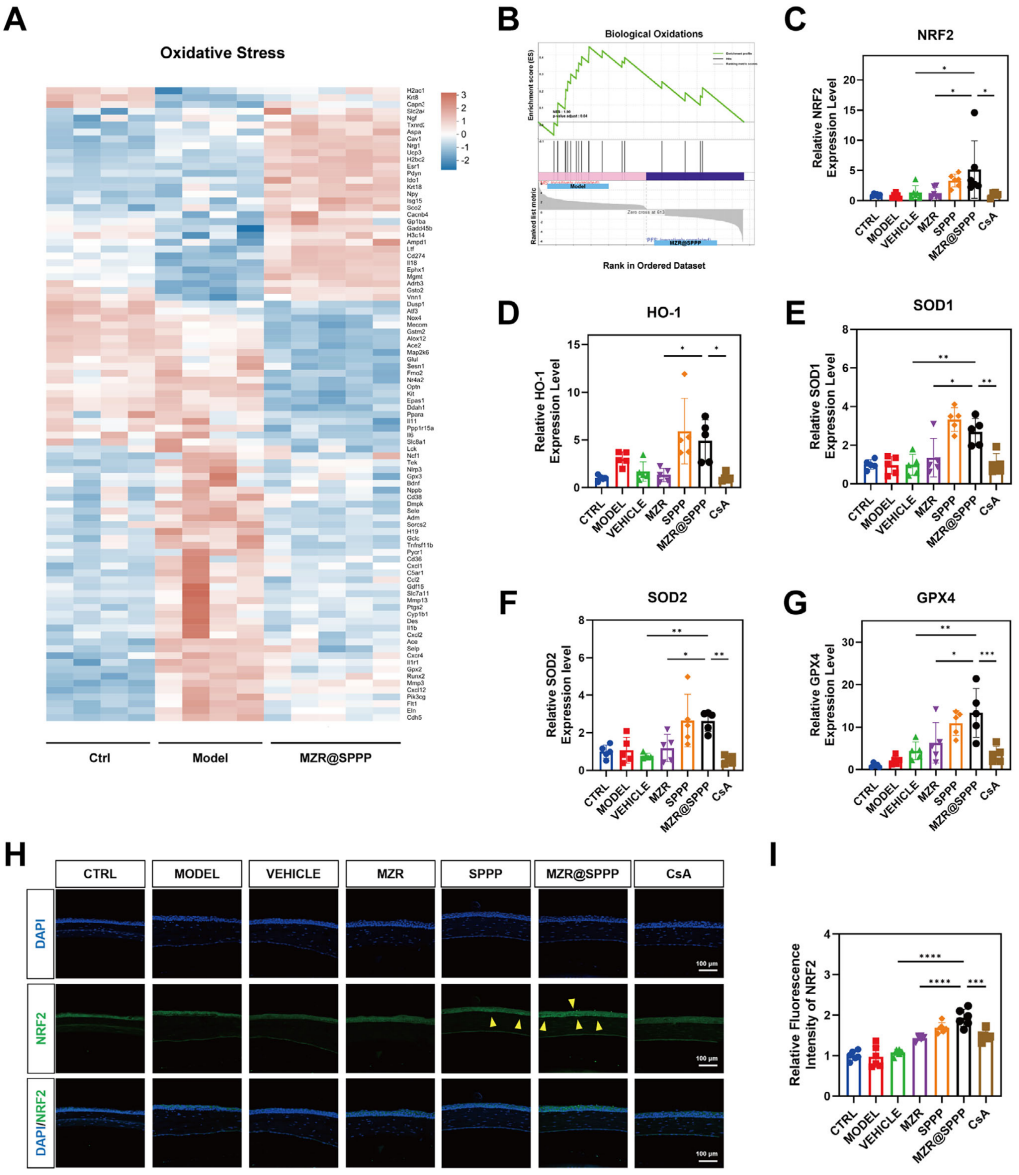
Antioxidant Capacity of MZR@SPPP Hydrogel in DED. (A) A heatmap visualizing the DEGs associated with oxidative stress. (B) GSEA of oxidative stress‐related pathways comparing Model and MZR@SPPP groups. The gene expression levels of NRF2 (C), HO‐1 (D), SOD1 (E), SOD2 (F), and GPX4 (G) in corneal tissues (n = 5). Immunofluorescence images of NRF2 (H) and quantitative analysis of the average fluorescence intensity (I) (n = 6). Yellow arrow: NRF2‐positive cells. Data are presented as mean ± SD. *p < 0.05; **p < 0.01; ***p < 0.001, ****p < 0.0001.

Inflammation plays a crucial role in the pathogenesis of DED, involving the dysregulated expression of various inflammatory cytokines and matrix metalloproteinases [73]. Elevated levels of pro‐inflammatory mediators, including IL‐1β, IL‐6, and TNF‐α, were identified in the tears of dry eye patients, compared to healthy individuals [74]. These cytokines promote immune cell recruitment, amplify the release of inflammatory factors, and mediate tissue damage through pathways including NF‐κB and MAPK. Given the pivotal role of inflammation in the pathogenesis of DED, anti‐inflammatory therapies have been incorporated as important treatment modalities [75]. Accordingly, the anti‐inflammatory capacity of MZR@SPPP in vivo was evaluated. Upon RNA‐seq analysis, MZR@SPPP significantly suppressed the expression of genes associated with inflammatory responses (Figure 10A) and downregulated interleukin‐mediated signaling pathways (Figure 10B). In the MZR@SPPP group, immunofluorescence staining showed a marked reduction in matrix metalloproteinase‐9 (MMP‐9) expression in the corneal epithelium (Figure 10C,D). Consistently, qRT‐PCR analysis confirmed that MZR@SPPP downregulated the mRNA levels of pro‐inflammatory mediators in corneal tissues, including MMP‐9, IL‐1β, IL‐6, and IFN‐γ (Figure 10E–H). Furthermore, WB analysis validated the downregulatory effect of MZR@SPPP on MMP‐9 and matrix metalloproteinase‐3 (MMP‐3) (Figure 10I–L). Specifically, MZR@SPPP suppressed the MAPK signaling pathway revealed by RNA‐seq (Figure 10M), which was supported by WB showing inhibited phosphorylation of p38 (Figure 10N,O). These results collectively suggested that MZR@SPPP alleviated corneal inflammation in DED through downregulation of key inflammatory cytokines and matrix metalloproteinases, as well as suppression of the MAPK signaling pathway (Figure 10P).

**FIGURE 10:**
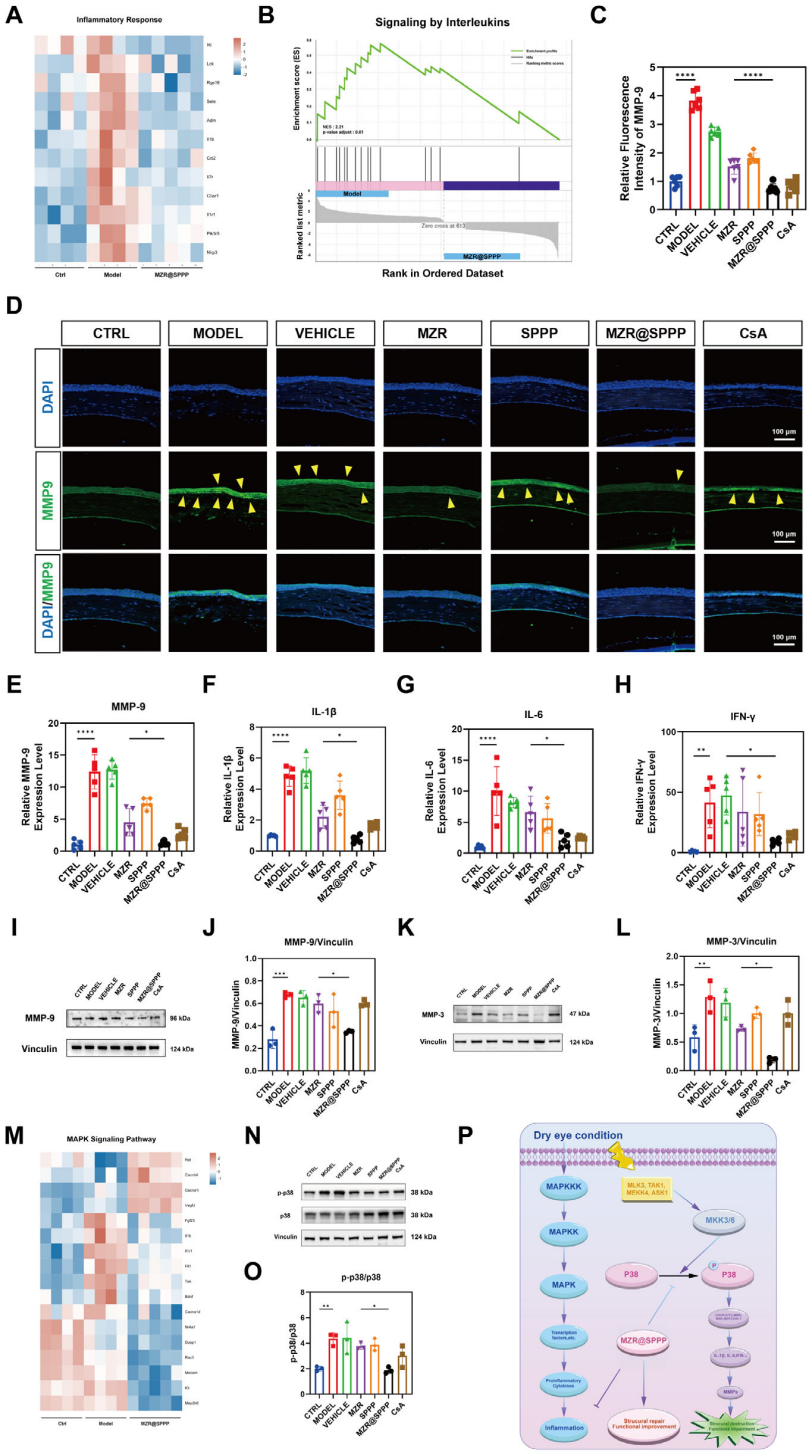
Anti‐inflammatory Capacity of MZR@SPPP Hydrogel in DED Through Suppression of MAPK Signaling Pathway. (A) A heatmap visualizing the DEGs associated with inflammatory response. (B) GSEA analysis of the DEGs involved in the signaling by interleukins, comparing Model and MZR@SPPP groups. MMP‐9 staining images in corneas (D) (Yellow arrow: MMP‐9‐positive cells) and the quantitative analysis of the average fluorescence intensity (C) (n = 6). Gene expression levels of MMP‐9 (E), IL‐1β (F), IL‐6 (G), and IFN‐γ (H) in corneas (n = 5). The WB image (I) and quantitative analysis (J) of MMP‐9 protein expression (n = 3). The WB image (K) and quantitative analysis (L) of MMP‐3 protein expression (n = 3). (M) A heatmap showing the DEGs involved in the MAPK signaling pathway. The WB image (N) and quantitative analysis (O) of p‐p38/p38 protein expression (n = 3). (P) A schematic diagram illustrating the potential anti‐inflammatory mechanism of MZR@SPPP in DED treatment. Data are presented as mean ± SD. *p < 0.05; **p < 0.01; ***p < 0.001, ****p < 0.0001.

Previous studies have indicated that resident macrophages in the cornea and conjunctiva tend to shift toward the inflammatory M1 phenotype under DS conditions, contributing to sustained inflammatory responses and epithelial damage [76]. Therefore, we investigated whether MZR@SPPP could mitigate these pathological changes in the ocular surface under dry eye conditions. The immunofluorescence staining for the macrophage marker F4/80 revealed significant macrophage infiltration and activation in the model group, which was markedly suppressed by MZR@SPPP treatment (Figure 11A,E). Furthermore, staining for the M1 markers iNOS and CD86 indicated a pronounced shift toward the M1 phenotype in the model group, an effect that was inhibited by MZR@SPPP (Figure 11B,C,F,G). And CD206 staining, which indicated the differentiation of M2 direction, suggested MZR@SPPP promoted M2 polarization (Figure 11D,H). RNA‐seq analysis revealed that MZR@SPPP exerted an inhibitory effect on the TNF signaling pathway (Figure 11I). Consistently, MZR@SPPP treatment downregulated the gene expression level of TNF‐α (Figure 11J).

**FIGURE 11:**
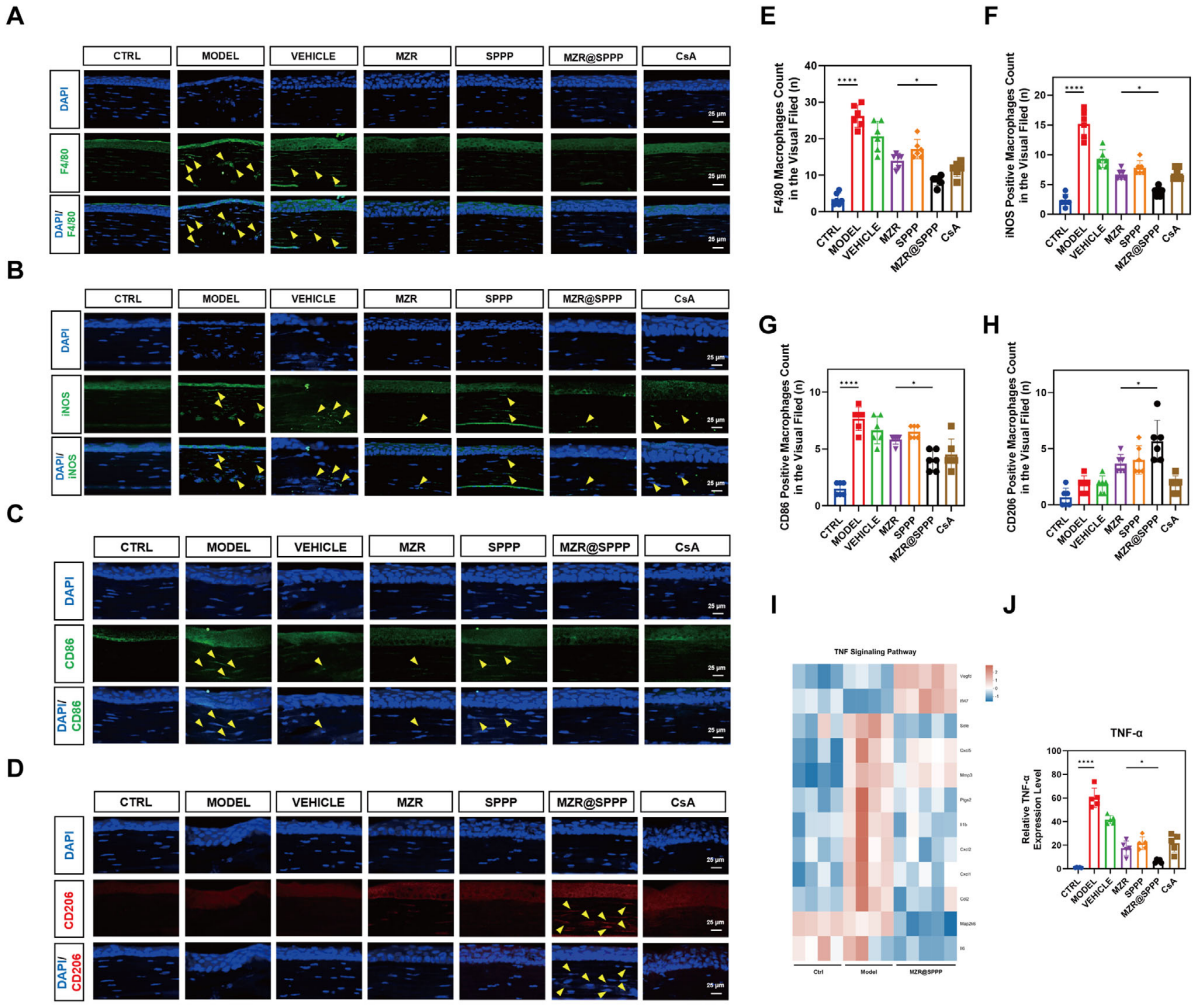
Anti‐inflammatory Capacity of MZR@SPPP Hydrogel in DED Through Modulating Macrophage Polarization. Immunofluorescence images (A) (Yellow arrow: F4/80‐positive cells) and the quantitative analysis of the average fluorescence intensity (E) (n = 6) of F4/80 in corneas. Immunofluorescence images (B) (Yellow arrow: iNOS‐positive cells) and the quantitative analysis of the average fluorescence intensity (F) (n = 6) of iNOS in corneas. Immunofluorescence images (C) (Yellow arrow: CD86‐positive cells) and the quantitative analysis of the average fluorescence intensity (G) (n = 6) of CD86 in corneas. Immunofluorescence images (D) (Yellow arrow: CD206‐positive cells) and the quantitative analysis of the average fluorescence intensity (H) (n = 6) of CD206 in corneas. (I) A heatmap presenting the DEGs involved in the TNF signaling pathway. (J) The gene expression level of TNF‐α in corneas (n = 5). Data are presented as mean ± SD. *p < 0.05; ****p < 0.0001.

Macrophages are the central regulators of the immune microenvironment, and the dynamic balance of their polarization between the pro‐inflammatory M1 phenotype and the anti‐inflammatory M2 phenotype is essential for maintaining immune homeostasis of the ocular surface. Under dry eye conditions, pro‐inflammatory cytokines such as TNF‐α and IL‐6 are present in the local microenvironment and promote macrophage polarization toward the M1 phenotype. These M1 macrophages, in turn, release additional inflammatory mediators that amplify inflammatory signaling and contribute to the persistence of epithelial damage on the ocular surface [76]. Consequently, there is growing interest in targeting ocular surface macrophages in the development of novel therapeutics for DED. Studies demonstrate that reprogramming macrophages from M1 to M2 phenotype can reverse corneal epithelial defects, restore goblet cell function, enhance tear secretion, and alleviate neural abnormalities in DED [77, 78, 79]. Importantly, innate immune activation precedes adaptive immune involvement in DED pathogenesis, positioning macrophages as an upstream intervention target [80]. In this context, MZR@SPPP modulates the ocular surface immune microenvironment by restraining pro‐inflammatory macrophage responses while promoting anti‐inflammatory polarization, thereby interrupting the inflammatory amplification cycle and supporting tissue repair at earlier stages of disease development.

Beyond macrophage regulation, MZR may also exert immunosuppressive effects through its established actions on adaptive immune cells. MZR is a purine synthesis inhibitor that preferentially suppresses lymphocyte proliferation, particularly T‐cell expansion and activation, making T cells a potential target of its immunosuppressive activity. In the present study, however, we primarily focused on the integrated effects of MZR@SPPP on oxidative stress and ocular surface inflammation, with particular emphasis on macrophage regulation. Although the observed anti‐inflammatory and immunomodulatory effects are consistent with the established pharmacological activity of MZR, the direct effects of MZR@SPPP on T cell responses were not evaluated. This represents a limitation of the present study. Future studies incorporating T‐cell subset profiling and functional analyses will be necessary to further clarify the contribution of adaptive immune regulation to the therapeutic effects of MZR@SPPP in DED.

The pathogenesis of DED involves oxidative stress, inflammation, and immune‐mediated damage on the ocular surface, resulting in ocular surface microenvironment imbalance [6]. The multifactorial etiology and pathogenic complexity of the disease make single‐target therapeutic strategies insufficient to comprehensively intervene in the disease process. Current clinical management often involves combinations of various pharmacological classes. However, such multi‐drug regimens present increased medication burden and may involve potential complex drug–drug interactions. The independent actions of individual components do not necessarily ensure synergistic therapeutic effects. In this context, the multi‐block copolymer SPPP functions not merely as a drug carrier but also as an active therapeutic biomaterial. By integrating ROS‐responsive, thermosensitive, mucoadhesive, sustained‐release, antioxidant, anti‐inflammatory, and immunoregulatory properties into a single formulation, MZR@SPPP represents a shift from conventional multi‐drug combinations toward an all‐in‐one multifunctional therapeutic strategy (Figure 12).

**FIGURE 12:**
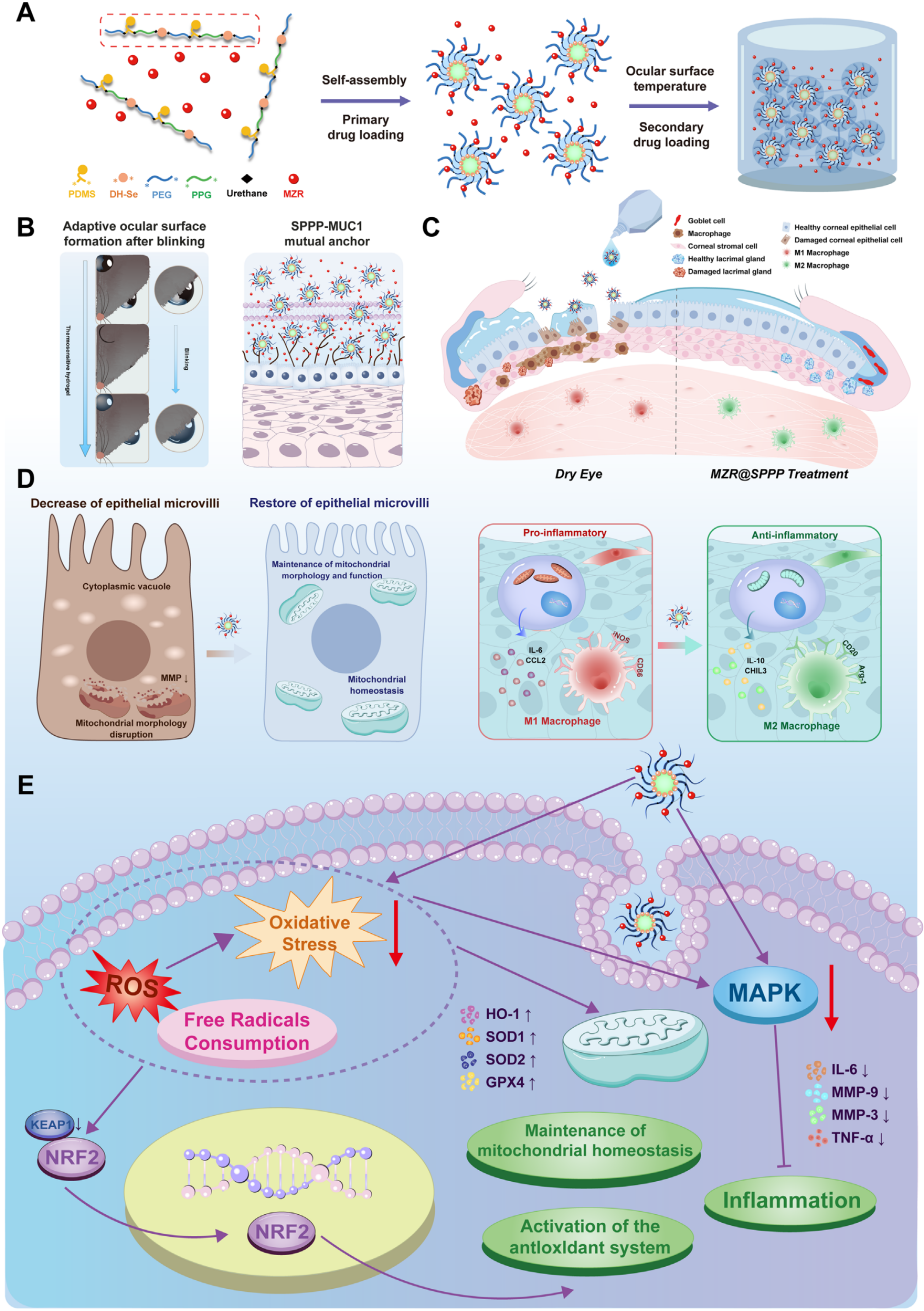
Schematic Illustration of Potential Therapeutic Mechanisms of MZR@SPPP in DED. (A) Drug loading process of MZR@SPPP hydrogel. (B) Thermosensitive gelation and adaptive layer formation of the hydrogel, and the underlying adhesion mechanism via SPPP and MUC1 interaction. (C) Therapeutic effects on the ocular surface microenvironment. (D) Regulatory effects on ocular surface epithelial cells and macrophages. (E) The molecular basis for the therapeutic efficacy of MZR@SPPP.

Importantly, oxidative stress and inflammation are closely interconnected in DED and can form a self‐amplifying pathological cycle. Excessive ROS accumulation can impair mitochondrial homeostasis and promote inflammatory activation, whereas sustained inflammation may further aggravate oxidative damage and epithelial dysfunction. Within this context, the therapeutic effects of MZR@SPPP likely arise from the complementary functions of its two components. The ROS‐responsive SPPP component reduces excessive ROS and helps restore the oxidative microenvironment, thereby potentially limiting oxidative stress‐driven inflammatory amplification. Meanwhile, ROS‐triggered MZR release provides downstream immunomodulatory effects by suppressing inflammatory responses and promoting an anti‐inflammatory macrophage phenotype. Thus, SPPP and MZR may act in a complementary manner to simultaneously target oxidative and inflammatory dysregulation, potentially interrupting the vicious cycle between oxidative stress and inflammation and promoting ocular surface restoration. Nevertheless, the individual contributions and temporal sequence of these two components were not quantitatively determined in the present study, and their relative roles may vary depending on treatment duration and pathological processes. Future studies involving longitudinal analyses and formulation optimization are warranted to further elucidate the synergistic mechanisms of this multifunctional platform.

Our study presents several key innovations and advantages. First, the active drug ingredient MZR in the formulation is a water‐soluble immunosuppressive agent, which is likely to exhibit minimal irritation to the ocular surface compared to existing lipophilic agents such as CsA and FK506. MZR is known to exert its selective inhibitory effect on lymphocyte proliferation by targeting inosine monophosphate dehydrogenase (IMPDH) and guanosine monophosphate (GMP) synthetase, thereby suppressing the synthesis of guanine nucleotides. In addition, it has been reported that MZR has been reported to inhibit antigen uptake in dendritic cells [81], reduce macrophage infiltration [82], and modulate macrophage inflammatory activation [83, 84]. Its mechanism of action differs from that of CsA and FK506, offering a therapeutic alternative in clinical practice. Moreover, the nanocarrier SPPP represents one of the most distinctive innovations of this work, offering new research perspectives and technical pathways for the field of ocular drug delivery. SPPP exhibits intrinsic antioxidant and mucoadhesive properties. Its antioxidative activity enables a synergistic effect with MZR in the treatment of DED, while its mucoadhesive capacity promotes a sustained drug release profile, thereby reducing the frequency of administration. In addition, this drug system delivers a synergistic therapeutic strategy through integrated antioxidant and anti‐inflammatory actions, mitochondrial function preservation, and immune microenvironment modulation, offering a multi‐targeted treatment method for DED that significantly improves overall therapeutic outcomes. Furthermore, the drug system demonstrates excellent biosafety, showing minimal toxicity in both in vitro cellular safety assessments and in vivo tissue compatibility studies. More importantly, MZR@SPPP demonstrated therapeutic effects comparable to the commercial CsA in promoting corneal epithelial repair, and outperformed CsA in enhancing tear secretion in a dry eye mouse model. Given its unique mechanism of action, excellent aqueous solubility, and minimal irritancy, MZR@SPPP represents a promising and valuable addition to the current armamentarium of ophthalmic immunosuppressive agents.

## 3 Conclusion

In conclusion, we have successfully developed a multifunctional copolymer, SPPP, which addresses the limitations of small‐molecule hydrophilic drugs in ocular delivery. By integrating intrinsic ROS‐scavenging capabilities and MUC1‐targeted mucoadhesion, the SPPP platform functions synergistically with the encapsulated immunosuppressant MZR to disrupt the vicious cycle of oxidative stress and inflammation in DED. This study presents an innovative approach that combines ROS‐responsive, mucoadhesive, thermosensitive, antioxidant, and anti‐inflammatory properties, offering a safe, effective, and gentle therapeutic option for complex multifactorial conditions such as DED. Further preclinical and clinical investigations are warranted to fully elucidate the therapeutic profile of MZR@SPPP and validate its efficacy in improving patient outcomes and quality of life. This study not only offers a highly efficacious, safe, and convenient nanotherapeutic strategy for dry eye but also highlights the immense potential of actively therapeutic biomaterials in treating complex multifactorial ocular surface diseases.

## 4 Experiment Section

### 4.1 Materials

PDMS, Se (99.5%), PEG (Mn = 2000), polypropylene glycol (PPG) (Mn = 2050), 1,6‐hexamethylene diisocyanate (HDI) (98%), dibutyltin dilaurate (DBT) (95%), acetonitrile (99.5%), CDCl_3_ (99.8 atom % D), pluronic F127 (PEO100PPO70PEO100), MZR, avertin, fluorescein sodium, and the goat anti‐rabbit immunoglobulin G (1:10 000; A0545) were purchased from Sigma‐Aldrich (Saint Louis, MO, USA). Anhydrous toluene, isopropanol, diethyl ether, hexane, methanol, and tetrahydrofuran were purchased from Acros Organics (Geel, Belgium). Phosphoric acid (85%–90%) was purchased from Macklin (Shanghai, China). Dulbecco's modified Eagle medium/nutrient mixture F‐12 was purchased from HyClone (Logan, UT, USA). Fetal bovine serum (FBS) was bought from Gibco (Eugene, OR, USA). Penicillin‐streptomycin solution, trypsin/0.25% ethylenediaminetetraacetic acid (EDTA), recombinant human insulin, and bovine serum albumin (BSA) were purchased from Yeasen (Shanghai, China). Recombinant human epidermal growth factor (hEGF) (C029) was obtained from Novoprotein (Shanghai, China). RAW 264.7 cell complete medium was purchased from Procell (Wuhan, China). PBS, Tween‐80, Rho, paraformaldehyde (4%), and PAS staining kit were purchased from Solarbio (Beijing, China). The amine coupling kit and Series S sensor chip CM5 were bought from Cytiva (Marlborough, MA, USA). Scopolamine hydrobromide (MB5860) was purchased from Meilubio (Dalian, China). Phenol red cotton threads were bought from Zone‐Quick (Yokota, Tokyo, Japan). Carbomer eye gel was procured from Bausch & Lomb (Rochester, NY, USA). The H&E staining kit was purchased from Auragene (Changsha, China). ROS assay kit (S1105S), total antioxidant capacity assay kit with a rapid ABTS method (S0121), total antioxidant capacity assay kit with FRAP method (S0116), ascorbic acid, one‐step TUNEL apoptosis assay kit (C1086), enhanced mitochondrial membrane potential assay kit with JC‐1 (C2003S), and Mito‐Tracker Red CMXRos (C1049B) were purchased from Beyotime (Nanjing, China). Anti‐KEAP1 antibody (1:200, A139729), anti‐KRT10 (1:200, ab76318), anti‐KRT12 (1:200, ab185627), and Live and Dead Cell Assay Kit (ab115347) were purchased from Abcam (Cambridge, UK). Anti‐NRF2 antibody (1:200/1:1000, A3577), anti‐TOMM20 (1:200, A26783), anti‐iNOS (1:200, A3774), anti‐IL‐6 (1:1000, A0286), anti‐Arg‐1 (1:200, A25808), anti‐MMP‐9 (1:200/1:1000, A0289), anti‐MMP‐3 (1:1000, A1202), anti‐phospho‐p38 MAPK (1:1000, A14401), anti‐p38 MAPK (1:1000, A0227), anti‐Vinculin (1:1000, A2752), and anti‐F4/80 (1:200, A1256) antibodies, along with CCK‐8 (RM02823) and ELISA kits (RK00027, RK00006, and RK00016), were procured from ABclonal (Wuhan, China). Anti‐CD86 (1:200, PA5‐114995), anti‐CD206 (1:200, MA5‐16871), Alexa Fluor 488‐conjugated donkey anti‐goat IgG (1:300, A11055), and Alexa Fluor 488‐conjugated donkey anti‐rabbit IgG (1:300; A21206) antibodies were bought from Invitrogen (Carlsbad, CA, USA). Anti‐CD86 (1:200, A17199A) and anti‐CD206 (1:200, C068C2) antibodies were obtained from BioLegend (San Diego, CA, USA). 4′,6‐diamidino‐2‐phenylindole (DAPI) (H‐1200) was procured from Vector (Burlingame, CA, USA). Tropicamide phenylephrine eye drops were purchased from Santen (Osaka, Japan). Commercial CsA, Zirun, was obtained from Xinqi Pharmaceutical (Shenyang, China). For reverse transcription and quantitative polymerase chain reaction (qPCR), TRIzol reagent (15596026) was purchased from Invitrogen (Carlsbad, CA, USA). HiScript III All‐in‐one RT SuperMix Perfect for qPCR and Taq Pro Universal SYBR qPCR Master Mix were purchased from Vazyme (Nanjing, China). The reagents used for WB, including the radioimmunoprecipitation assay (RIPA) buffer (89901), the proteinase and phosphatase inhibitor cocktail (78442), the bicinchoninic acid (BCA) assay kit (23225), the PageRuler prestained protein ladder, and the Restore PLUS Western Blot Stripping Buffer (46430) were obtained from Thermo Fisher Scientific (Waltham, MA, USA). Polyvinylidene difluoride (PVDF) membranes were procured from Millipore (Billerica, MA, USA). The enhanced chemiluminescent reagent was bought from NCM Biotech (Suzhou, China).

### 4.2 Synthesis of SPPP Copolymers

SPPP was derived from PDMS, Se, PEG, and PPG at a feed ratio of 1:2:2:1, with HDI and DBT as the catalyst and the chain extender. Specifically, starting materials (13.079 g) including PDMS (10 g, 0.5 mmol), Se (0.079 g, 1 mmol), PEG (2 g, 1 mmol), and PPG (1 g, 0.5 mmol) were charged into a round‐bottomed flask and dissolved in anhydrous toluene (100 mL). Traces of water were removed by azeotropic distillation in a rotary evaporator (R‐210, Buchi, Flawil, Switzerland) until the volume was reduced to 10 mL; this procedure was repeated twice. Under an argon atmosphere, the mixture was stirred at 110°C, and HDI (0.84 mL, 5.2 mmol) and two drops of DBT were injected into the flask. The reaction proceeded for 24 h, with additional anhydrous toluene (10 mL) added as needed to maintain stirring when the mixture became too viscous. The crude product was precipitated in hexane‐diethyl ether (3:7 v/v), redissolved in isopropanol, and purified by dialysis against deionized water for 3 days. SPPP was finally obtained by freeze‐drying (Scientz‐10N/A, Scientz, Ningbo, China) for 3 days.

### 4.3 Molecular Characterization

FT‐IR spectra were collected using a Spectrum 2000 FT‐IR spectrophotometer (Frontier, PerkinElmer, MA, USA) at room temperature. The copolymers were mixed with potassium bromide and tableted, undergoing 32 scans in the wavenumber range of 4000–400 cm^−1^ resolution of 4 cm^−1^.

^1^H and ^13^C NMR spectra were obtained using a 500‐MHz NMR spectrometer (JES‐FA200, JEOL, Tokyo, Japan) at room temperature. The chemical shift was referenced to the solvent peak of CDCl_3_ at 7.3 ppm.

The molecular weight characteristics were determined by GPC (LC‐2050, Shimadzu, Kyoto, Japan) using PEG/polyethylene oxide (PEO) standards for calibration. Mn, Mw, and Đ were calculated based on the GPC analysis.

UV–vis spectra were recorded using a UV–vis spectrophotometer (UV‐3600 Plus, Shimadzu, Japan) over the wavelength range of 200–800 nm at a polymer concentration of 20 wt%.

### 4.4 Thermal Analysis

TGA was carried out on a Q500 TGA analyzer (TA Instruments, New Castle, DE, USA) under a nitrogen atmosphere. The temperature was elevated from room temperature to 800°C at a rate of 20°C/min. DSC was performed on a Q100 photo‐DSC analyzer (TA Instruments, New Castle, DE, USA) using indium for calibration. Two heating‐cooling cycles in the range of −80°C to 200°C were run at a heating or cooling rate of 20°C/min, and data from the second cycle were used for analysis.

### 4.5 Zeta Potential and Particle Size Analysis

Zeta potentials of SPPP and mucin solutions, and the particle size and distribution of SPPP and MZR@SPPP solutions were measured by DLS on a Nano ZetaSizer System (Nano‐ZS, Malvern Instruments, Malvern, UK).

### 4.6 Scanning Electron Microscope

SPPP and MZR@SPPP hydrogels were subjected to ultra‐low temperature vacuum freeze‐drying and prepared for SEM observation. To evaluate ROS‐responsive structural changes, SPPP hydrogels were incubated in PBS or PBS containing H_2_O_2_ (500 µM) at 37°C and collected at predetermined time points (0, 3, 6, and 12 h). Samples were freeze‐dried, sectioned, and then coated with gold particles using an ion sputter coater (Q150RS, Quorum Technologies, Lewes, UK). SEM images were acquired using a Stereoscan 260 microscope (Cambridge Instruments, Cambridge, UK) at an acceleration voltage of 20 kilovolts.

### 4.7 Molecular Docking and Molecular Dynamics Simulation

The amino acid sequence of MUC1 was retrieved from the UniProt database (https://www.uniprot.org/). The specifical amino acid sequence was adopted to conduct the simulation process. For molecular docking, water molecules and irrelevant heteroatoms were removed using UCSF Chimera (version 1171, RBVI, San Francisco, CA, USA) to preserve the protein structure alone. Protein atomic charges were calculated using the Amber14 SB force field, and amino acid pKa values were determined and assigned under neutral conditions using the H++ online tool. Modified peptide structures were generated using Chimera. Conformation sampling was optimized using the AMBER molecular force field to obtain low‐energy conformations. Austin model 1‐bond charge correction local charges were assigned using UCSF Chimera. The optimal binding site was predicted using SiteMap software (version 55, Schrödinger, New York, USA). For molecular dynamics simulation, 3D protein structures were constructed using AlphaFold2 (version 232, DeepMind, London, UK). The protein dimer cross‐linked structure was constructed using Avogadro software (version 1910, Kitware, New York, USA, 2023). Methacrylic anhydride crosslinking modifications were performed according to the lysine residues exposed to the protein surface. The Gromacs 5.1.5 open‐source software package (https://www.gromacs.org/) was employed for the simulations, with the system configured in a confined environment at 300 K, pH 7.4, and a pressure of 1 bar. The transferable intermolecular potential 3‐point (TIP3P) water molecule was adopted, and the system charge was neutralized with sodium chloride solvent. Following system construction, energy minimization was performed using the steepest descent algorithm applied to all atoms. The system underwent 200 nanoseconds (ns) molecular dynamics simulations with a 2 femtoseconds (fs) time step. After completion of all simulations, structural parameters including radius of gyration (Rg), solvent accessible surface area (SASA), and root mean square deviation (RMSD), and root mean square fluctuation (RMSF) were calculated.

### 4.8 Rheological Analysis

Rheological measurements were performed on a Discovery DHR‐3 hybrid rheometer (TA Instruments, New Castle, DE, USA) with parallel‐plate geometry. Both temperature ramps (from low temperature to body temperature) and temperature sweeps (4°C–80°C at a heating rate of 5°C/min) were conducted at a fixed strain of 1% and frequency of 1 Hz.

### 4.9 Surface Plasmon Resonance

SPR analysis was performed on a BIAcore‐T200 biomolecular interaction analyzer (Cytiva, Marlborough, MA, USA) to analyze the interactions between MUC1 and polymers. MUC1 was dialyzed in ice‐cold PBS for 48 h to remove buffer salts, followed by lyophilization. Acetate buffers of varying pH were prepared to screen for optimal protein coupling conditions. MUC1 was immobilized on a CM5 sensor chip via amine coupling to a level of approximately 7000 response units (RU). SPPP and F127 were serially diluted in PBS to final concentrations of 50, 25, 12.5, 6.25, 3.125, 1.5625, and 0.78125 µM. Each concentration was injected over the chip at a flow rate of 30 µL/min with a 120‐s contact time and a 450‐s dissociation phase. The chip surface was regenerated with pH 2.5 glycine buffer and washed with PBS between cycles. Experiments were performed at 25°C. Binding data were analyzed using BIAcore T200 Evaluation Software (version 2.0, GE Healthcare, Piscataway, NJ, USA) and fitted to a 1:1 binding model to calculate the K_D_ value.

### 4.10 Preparation and Physicochemical Characterization of MZR@SPPP

To prepare the SPPP hydrogel, a certain weight of SPPP copolymer was dissolved in PBS, placed in a magnetic stirrer to remove bubbles, and formed a uniform mixed solution. MZR (50 µg/mL) was physically incorporated into the SPPP solution (20%) through direct mixing overnight, whereby a predetermined amount of MZR was introduced into the SPPP solution under low‐speed stirring. The mixture was sonicated using an ultrasonic homogenizer (Omni Sonic Ruptor 4000, OMNI International, Kennesaw, GA, USA) at a frequency of 20 kHz and 90% power to ensure homogeneous dispersion of MZR within the SPPP solution. The resulting formulation was subsequently used for further characterization and biological evaluation.

The short‐term storage stability of SPPP and MZR@SPPP formulations was preliminarily evaluated under different storage conditions. Briefly, the formulations were stored at 4°C for 0, 7, 14, and 22 days. At each time point, macroscopic images were recorded, and particle size and zeta potential were measured by DLS. On day 22, the MZR content in the MZR@SPPP formulation was quantified by HPLC.

### 4.11 In Vitro Drug Release Experiment and High‐performance Liquid Chromatography

MZR@SPPP solution (500 µL) was placed in a dialysis tube (MW3000, SALMART, Shanghai, China) and incubated at 34.5°C in a constant‐temperature water bath to form a hydrogel. Then, PBS (50 mL) was gently layered on top of the hydrogel to serve as the release medium. The tube was vertically placed in a constant‐temperature shaker maintained at 34.5°C and 50 rpm. At predetermined time intervals (0, 0.5, 1, 2, 4, 6, 8, 12, 24, 48, and 72 h), the upper release medium was withdrawn and replaced with an equal volume of fresh PBS. This process was repeated until the hydrogel was fully degraded. The drug release experiment for the MZR@SPPP hydrogel in H_2_O_2_ solution (500 µM) was conducted following a similar procedure. At predetermined time intervals (0, 0.5, 1, 2, 4, 8, and 12 h), the upper release medium was withdrawn and replaced with an equal volume of fresh H_2_O_2_ solution.

The collected samples were stored at −20°C until analysis. The concentration of MZR was determined by HPLC to generate drug release profiles. Chromatographic separation was performed on a ZORBAX SB‐C18 column (5 µm, 4.6 × 150 mm, Agilent Technologies, Santa Clara, CA, USA) using a mobile phase of 0.1% phosphoric acid in water/acetonitrile (90:10, v/v) at a flow rate of 1 mL/min. Detection was carried out at 279 nm, the column temperature was maintained at 25°C, and the injection volume was 10 µL. A stock solution of MZR was prepared by dissolving an accurately weighed quantity of the standard powder in double‐distilled water to a final concentration of 5 mg/mL. For the calibration standards, this stock solution was subsequently serially diluted with the mobile phase to obtain concentrations ranging from 10 to 1600 µg/mL (10, 20, 50, 100, 200, 400, 800, and 1600 µg/mL). Each standard solution was then analyzed by HPLC, and a calibration curve was constructed by plotting the measured peak area against the corresponding concentration of MZR.

### 4.12 Pharmacokinetic Evaluation and High‐performance Liquid Chromatography‐Mass Spectrometry/Mass Spectrometry

Female New Zealand white rabbits were purchased from the Animal Center of Xiamen University (Xiamen, China). The animal protocols conformed to the Association for Research in Vision and Ophthalmology (ARVO) Statement for the Use of Animals in Ophthalmic and Vision Research. This research program was approved by the Experimental Animal Ethics Committee of Xiamen University. The rabbits were kept in a well‐ventilated environment at a humidity of 60 ± 3% and a temperature of 23°C–25°C. MZR and MZR@SPPP eye drops were applied to the outer one‐third of the lower conjunctival sac of the rabbits’ eyelids (50 µL per eye), and applied again after manually blinking three times and waiting for 30 s. The rabbits were euthanized at 1, 3, and 6 h post‐instillation. Rabbits were humanely euthanized via carbon dioxide (CO_2_) asphyxiation in accordance with American Veterinary Medical Association (AVMA) guidelines. Compressed CO_2_ was introduced into a sealed chamber at a controlled displacement rate to first induce unconsciousness and then ensure respiratory arrest. Death was confirmed by absence of respiration, corneal reflex, and cardiac activity. After rinsing the corneal surface with physiological saline, the aqueous humor and corneas were collected.

The processed corneal samples were ground, mixed with methanol, and vigorously agitated to facilitate drug extraction into the solvent. Following centrifugation, the supernatant was collected and concentrated under low temperature and vacuum conditions using a CentriVap system (Labconco, Kansas City, MO, USA). The resulting residues were redissolved in methanol. Finally, the prepared corneal samples, along with aqueous humor samples, internal standards (Table S1), and external standards (Table S2), were subjected to high‐performance liquid chromatography‐mass spectrometry/mass spectrometry (HPLC‐MS/MS) analysis for the quantification of MZR concentrations.

### 4.13 Precorneal Retention Evaluation

Stereo fluorescence microscopic imaging, in vivo imaging, and cryopreserved section analysis were performed to evaluate precorneal retention and permeation. For stereo fluorescence microscopic imaging, the mice were anesthetized with 1.25% avertin (250 mg/kg) and then instilled with free Rho (5 µL, 1 mM) in the left eyes and Rho@SPPP (5 µL, 1 mM equivalent to that of free Rho) in the right eyes. Fluorescence intensity changes were monitored using a stereo fluorescence microscopic imaging system (M165FC, Leica, Wetzlar, Germany) at 0, 10, 20, 30, 40, 50, 60, 90, 120, and 150 min. For in vivo imaging, identical Rho and Rho@SPPP eye drops were applied to the corneal surface of mice under isoflurane (2%) gas anesthesia (300 mL/min). Fluorescence intensity variations were recorded using an in vivo imaging system (IVIS Spectrum, PerkinElmer, Waltham, MA, USA) at 0, 30, 60, and 120 min (Ex/Em: 535 nm/600 nm). For cryopreserved section imaging, the same eye drops were administered to the corneal surface of mice under consciousness. Mice were euthanized 2 and 10 min post‐administration, soon after which their eyeballs were carefully removed and frozen in liquid nitrogen with optimum cutting temperature (4583, SAKURA, Torrance, CA, USA). Sections (5 µm thick) were prepared and then observed under a fluorescence microscope (DM2500, Leica, Wetzlar, Germany). The image intensity was quantified by ImageJ software (version 1.54 g, National Institutes of Health, Bethesda, MD, US).

### 4.14 Evaluation of Cellular Internalization Profiles

HCE cells were cultured on microscope cover glasses in 24‐well plates and cultured overnight, followed by incubation with Rho (1 mM) and Rho@SPPP (1 mM equivalent to that of free Rho) for 2 h. After three washes, cells on the glasses were stained with DAPI and visualized under a fluorescence microscope (DM2500, Leica, Wetzlar, Germany). Quantitative analysis was performed by measuring the average fluorescence intensity using ImageJ software (version 1.54 g, National Institutes of Health, Bethesda, MD, USA).

### 4.15 Anti‐Oxidative Effect Evaluation Using ABTS and FRAP

The total antioxidant capacity of MZR@SPPP was evaluated using the rapid ABTS and FRAP methods according to standard protocols. Ascorbic acid (100 µg/mL) was used as a positive control, with its antioxidant capacity defined as 100%. The antioxidant capacity of SPPP at various concentrations (50, 100, 250, 500, 1000, 2000, 5000 µg/mL) was calculated relative to the positive control.

### 4.16 In Vitro Assessment of Antioxidative Capacity, Mitochondrial Protective Effects, and Modulation of Macrophage Polarization

HCE cells (within 10 passages) were planted in a 96‐well plate and treated with H_2_O_2_ and different drugs (MZR, SPPP, and MZR@SPPP) for 6 h. The medium was aspirated, and the cells were washed with PBS following the treatment. Subsequently, the cells were incubated with CCK‐8 at 37°C for 2 h. The optical density (OD)‐value of each well was measured at 450 nm by a microplate reader (ELx800, BioTek, Winooski, VT, USA).

HCE cells were seeded in a 12‐well plate and treated with H_2_O_2_ and different drugs (MZR, SPPP, and MZR@SPPP) for 6 h. The assessment of ROS, apoptosis, MMP, and mitochondrial localization was conducted using DCFH‐DA staining, TUNEL assay, JC‐1 staining, and MitoTracker Red CMXRos staining, respectively, in accordance with standard protocols.

RAW264.7 cells (within 10 passages) were planted in a 6‐well plate, treated with LPS (100 ng/mL) and IFN‐γ (20 ng/mL) for M1 polarization, or IL‐4 (20 ng/mL) and IL‐13 (20 ng/mL) for M2 polarization for 24 h, and simultaneously exposed to the respective drug (MZR, SPPP, and MZR@SPPP). For flow cytometric analysis, cells were stained with FITC‐conjugated anti‐CD86 antibody or FITC‐conjugated anti‐CD206 antibody in the dark at room temperature for 15 min. The samples were tested on a flow cytometer (CytoFlex S, Beckman, CA, USA). For ELISA analysis, cell culture supernatants were quantified using mouse‐specific ELISA kits according to the manufacturer's instructions, and cytokine levels were calculated based on the corresponding standard curves.

### 4.17 In Vivo Dry Eye Model and Topical Drug Treatment

8‐ to 12‐week female C57BL/6 mice were purchased from the Animal Center of Xiamen University (Xiamen, China). The animal protocols conformed to the ARVO Statement for the Use of Animals in Ophthalmic and Vision Research. This research program was approved by the Experimental Animal Ethics Committee of Xiamen University. The mice were housed in well‐ventilated conditions with a temperature maintained at 23°C–25°C and relative humidity of 30 ± 3%. The dry eye model was established by inducing DS through subcutaneous injections of scopolamine hydrobromide (0.25 g/100 mL) administered four times daily for seven consecutive days, combined with continuous airflow exposure using small electric fans to enhance ocular surface drying. A BAC‐induced dry eye model was established by topical application of 0.2% BAC eye drops twice daily for 7 days. To minimize pain and stress, all subcutaneous injections were performed by trained researchers using skilled techniques to prevent secondary injury to the animals. In parallel, topical treatment (PBS, MZR, SPPP, MZR@SPPP, and CsA) was administered (5 µL per eye) twice daily for 7 days without anesthesia.

### 4.18 Examination of Dry Eye Manifestations

For tear secretion assessment, a phenol red thread was placed in the lower conjunctival fornix, and the mouse was briefly restrained to remain stationary for 15 s. Tear secretion rate was measured in millimeters by the distance of the red‐stained tear front. Each measurement was performed in triplicate, and the average value was recorded.

For corneal fluorescein staining, 1% sodium fluorescein was instilled onto the mouse ocular surface for 1 min, followed by 3 gentle manual blinks. The fluorescein staining images were captured under cobalt blue light using a digital slit‐lamp biomicroscope. Corneal fluorescein staining was graded according to the Chinese Consensus on Dry Eye Disease (2020): the cornea was divided into 4 equal quadrants, with each quadrant scored individually (Absent: 0; punctate staining ≤30 spots: 1; punctate staining >30 spots but non‐diffuse: 2; diffuse staining: 3). The total score was the sum of all quadrant scores (maximum: 12 points).

Tear film stability was evaluated by measuring TBUT. Briefly, fluorescein sodium was applied to the ocular surface, and the interval between the last blink and the appearance of the first dry spot was recorded under a slit‐lamp microscope. TBUT was measured three times for each eye, and the average value was calculated.

### 4.19 Histological Staining

The murine eyeballs, along with the periocular adnexa were dissected and fixed in 4% formaldehyde for 24 h. The fixed tissues were then processed into paraffin blocks. Paraffin‐embedded samples were cut into sagittal sections (5 µm thick) and stained using standard protocols for H&E and PAS, following the manufacturer's instructions. All slides were examined under a light microscope (Eclipse E400 equipped with a DS‐Fi1 camera, Nikon, Tokyo, Japan) for observation and image acquisition.

### 4.20 Immunofluorescence Staining

The sections were incubated overnight at 4°C with primary antibodies against KEAP1, KRT10, KRT12, NRF2, TOMM20, CD86, iNOS, CD206, Arg‐1, MMP‐9, or F4/80. On the following day, the sections were washed three times with PBS and then incubated with corresponding secondary antibodies for 1 h at room temperature in the dark. Finally, the sections were mounted with DAPI and imaged using a fluorescence microscope (DM2500, Leica, Wetzlar, Germany).

### 4.21 Quantitative Real‐Time Polymerase Chain Reaction

Total RNA was extracted from the cells or corneal tissues using TRIzol reagent and purified through chloroform extraction. An equivalent amount of RNA was reverse‐transcribed, and qRT‐PCR analysis was performed on a LightCycler 96 system (Roche, Mannheim, Germany). The thermal cycling protocol consisted of an initial denaturation at 95°C for 30 s, followed by 40 cycles of denaturation at 95°C for 10 s, and annealing/extension at 60°C for 30 s. Amplification specificity was confirmed by melting curve analysis. All reactions were performed in duplicate, and relative expression levels were calculated using the 2^–ΔΔCt^ method. The primers are provided in Table S3.

### 4.22 Western Blotting

Cell samples or corneal tissue samples were lysed in cold RIPA buffer supplemented with protease and phosphatase inhibitor cocktail. Protein concentrations were determined using a BCA assay kit. Equal amounts of protein were subjected to electrophoresis on 12% tricine gels and transferred to PVDF membranes. The membranes were blocked with 5% BSA for 2 h at room temperature, followed by incubation overnight with primary antibodies. After washing three times with Tris‐buffered saline containing 0.05% Tween‐20, the membranes were incubated for 1 h with corresponding secondary antibodies. Protein bands were visualized using an enhanced chemiluminescent reagent, and images were acquired with a ChemiDoc imaging system (Bio‐Rad, Hercules, CA, USA).

For sequential detection of different target proteins on the same membrane, the membrane was treated with stripping buffer for 10 min after initial imaging, washed, and blocked again. It was then sequentially incubated with the primary antibody against the subsequent target protein and the corresponding secondary antibody, followed by chemiluminescent detection as described above.

Band intensities were quantified using Image Lab software (version 5.2, Bio‐Rad). The intensity of each target protein band was normalized to that of the corresponding vinculin band from the matched sample lane on the same membrane to determine relative protein expression.

### 4.23 Safety Evaluation

In vitro, HCE cells were planted in a 96‐well plate and treated with MZR, SPPP, and MZR@SPPP at a range of concentrations for 12, 24, and 48 h. The medium was aspirated, and the cells were washed with PBS following the treatment. Subsequently, the cells were incubated with CCK‐8 at 37°C for 2 h. The OD value of each well was measured at 450 nm by an ELx800 Microplate Reader (Bio Tek Instruments, VT, USA). HCE cells were planted in a 12‐well plate, treated with 500 µg/mL SPPP and MZR@SPPP for 12 h, and stained with a Live and Dead Cell Assay Kit according to the manufacturer's instructions.

In vivo, mice were treated topically with SPPP or MZR@SPPP twice daily. For short‐term safety evaluation, mice were treated for 7 days, while extended safety evaluation was performed after repeated administration for 1 month. Prior to the examinations, anesthesia was induced via intraperitoneal injection of 1.25% avertin (250 mg/kg). Pupillary dilation was achieved using tropicamide phenylephrine eye drops, and the ocular surface was lubricated with carbomer eye gel to maintain corneal clarity during imaging. Ocular examinations, including slit‐lamp biomicroscopy, corneal confocal microscopy (Heidelberg Engineer, Heidelberg, Germany), OCT (Casia II, TOMEY, Nagoya, Japan), fundus photography (OPIMG‐L, OPTOPROBE, UK), and electroretinography (RetiMINE R System, AiErXi Medical Equipment Co., Ltd., Chongqing, China), were performed following standardized protocols. Following the 1‐month repeated administration, ocular tissues and major organs (heart, liver, spleen, lung, and kidney) were collected for H&E staining. Serum samples were collected for hepatic and renal function analysis to evaluate systemic toxicity.

### 4.24 Statistical Analysis

The data obtained from the experiments were processed using GraphPad Prism software (version 7.0, GraphPad, San Diego, CA, USA). Continuous variables with a normal distribution were presented as the mean ± SD. Categorical variables were summarized as numbers and percentages (%). Differences between two groups for continuous variables were assessed using an unpaired Student's *t*‐test, while one‐way analysis of variance (ANOVA) was employed for comparisons across more than two groups. Associations between categorical variables were evaluated with the Chi‐square (χ^2^) test. A two‐sided *p*‐value of less than 0.05 was considered statistically significant. Statistical significance of *p* values was indicated as *: *p* < 0.05, **: *p* < 0.01, ***: *p* < 0.001, and ****: *p* < 0.0001.

**Abbreviations**

Abbreviations are provided in Table S4.

## Author Contributions

**Danyi Qin**: conceptualization, methodology, data curation, writing – review and editing, writing – original draft, formal analysis. **Jiani Li**: conceptualization, methodology, writing – review and editing, formal analysis, data curation. **Panqin Ma**: conceptualization, methodology, writing – review and editing, formal analysis, data curation. **Shinan Wu**: methodology, writing – review and editing, data curation, formal analysis. **Wenying Guan**: methodology, writing – review and editing, formal analysis, data curation. **Yimin Huang**: methodology, formal analysis, project administration, writing – review and editing. **Ke Feng**: methodology, writing – review and editing, project administration, formal analysis. **Mingyan Wei**: methodology, writing – review and editing, project administration, formal analysis. **Zixuan Yang**: methodology, writing – review and editing, project administration, formal analysis. **Shujia Guo**: methodology, writing – review and editing, project administration, formal analysis. **Caihong Huang**: supervision, formal analysis, project administration, funding acquisition, writing – review and editing, investigation, conceptualization. **Yun‐Long Wu**: writing – review and editing, project administration, formal analysis, data curation, investigation, conceptualization. **Yi Han**: investigation, conceptualization, funding acquisition, project administration, formal analysis, data curation, writing – review and editing. **Zuguo Liu**: data curation, project administration, formal analysis, writing – review and editing, funding acquisition, investigation, conceptualization.

## Ethics Approval Statement

This study was approved by the Ethics Committee of Xiamen University (**XMULAC20220258**, Xiamen, Fujian, China) and was performed following the Association for Research in Vision and Ophthalmology (ARVO) Statement for the Use of Animals in Ophthalmic and Vision Research.

## Conflicts of Interest

All authors declared that the research was conducted in the absence of any commercial or financial relationships that could be construed as a potential conflict of interest.

## Declaration on Artificial Intelligence in Figure Preparation

No artificial intelligence tools were used in figure preparation.

## Supporting information




**Supporting File**: advs78106‐sup‐0001‐SuppMat.docx.

## Data Availability

The data that support the findings of this study are openly available in the China National Center for Bioinformation (CNCB) at https://ngdc.cncb.ac.cn/gsa/s/M3cqj9fa, reference number CRA050144.
