# Supplementary material for: A ROS‐Responsive and Mucoadhesive Thermosensitive Hydrogel Encapsulated with Mizoribine for Synergistic Dry Eye Therapy
Source: Adv Sci (Weinh). 2026 Oct 6:e78106. Online ahead of print. doi: 10.1002/advs.78106 (PMC13643235; doi:10.1002/advs.78106)
Supplement: Supplementary file 1 [file ADVS-9999-e78106-s001.docx]

**A ROS-Responsive and Mucoadhesive Thermosensitive Hydrogel Encapsulated with Mizoribine for Synergistic Dry Eye Therapy**

**AUTHOR NAMES**

Danyi Qin^1‡^, Jiani Li^1‡^, Panqin Ma^2‡^, Shinan Wu^1^, Wenying Guan^1^, Yimin Huang^1^, Ke Feng^1^, Mingyan Wei^2^, Zixuan Yang^1^, Shujia Guo^2^, Caihong Huang^1*^, Yun-Long Wu^3*^, Yi Han^1, 2*^, Zuguo Liu^1, 2*^

**AUTHOR ADDRESS**

^1^Xiamen University affiliated Xiamen Eye Center; Fujian Provincial Key Laboratory of Ophthalmology and Visual Science; Fujian Engineering and Research Center of Eye Regenerative Medicine; Eye Institute of Xiamen University; School of Medicine, Xiamen University, Xiamen, Fujian 361005, China

^2^Department of Ophthalmology, The First Affiliated Hospital of University of South China, Hengyang Medical School, University of South China, Hengyang, Hunan 421001, China

^3^Fujian Provincial Key Laboratory of Innovative Drug Target Research and State Key Laboratory of Cellular Stress Biology, School of Pharmaceutical Sciences, Xiamen University, Xiamen, Fujian 361005, China

^‡^These authors contributed equally to this work.

***Corresponding author.**

**Zuguo Liu, Ph.D., M.D.**

Xiamen University Affiliated Xiamen Eye Center, Fujian Provincial Key Laboratory of Ophthalmology and Visual Science, Fujian Engineering and Research Center of Eye Regenerative Medicine, Eye Institute of Xiamen University, School of Medicine, Xiamen University, Xiamen, Fujian 361005, China

Email: zuguoliu@xmu.edu.cn

**Yi Han, Ph.D., M.D.**

Department of Ophthalmology, The First Affiliated Hospital of University of South China, Hengyang Medical School, University of South China, Hengyang, Hunan 421001, China

Email: hanyi@usc.edu.cn; [hanyixmu@foxmail.com](mailto:hanyixmu@foxmail.com)

**Yun-Long Wu, Ph.D.**

School of Pharmaceutical Sciences, and Fujian Provincial Key Laboratory of Innovative Drug Target Research, Xiamen University, Xiamen, Fujian 361102, China.

Email: wuyl@xmu.edu.cn

**Caihong Huang, M.S.**

Xiamen University Affiliated Xiamen Eye Center, Fujian Provincial Key Laboratory of Ophthalmology and Visual Science, Fujian Engineering and Research Center of Eye Regenerative Medicine, Eye Institute of Xiamen University, School of Medicine, Xiamen University, Xiamen, Fujian 361005, China

Email: huangcaihong0327@126.com; huangcaihong@xmu.edu.cn

**Supplementary Figures**

**Figure S1. ¹H nuclear magnetic resonance (NMR) spectra of Polydimethylsiloxane (PDMS), poly(Se-PEG-PPG) (SPP), and poly(Se-PDMS-PEG-PPG) (SPPP).**

**Figure S2. ¹³C NMR spectra for PDMS, SPP, and SPPP, along with a collective display.**

**Figure S3. Size distribution of aqueous Mizoribine (MZR)@SPPP solution.**

**Figure S4. Representative scanning electron microscope (SEM) image of the MZR@SPPP hydrogel.**

**Figure S5. Macroscopic images of SPPP and MZR@SPPP formulations recorded on days 0, 7, 14, and 22 during storage at 4°C.**

**Figure S6. Particle sizes and zeta potentials of SPPP and MZR@SPPP formulations measured on days 0, 7, 14, and 22 during storage at 4°C.**

**Figure S7. MZR concentrations in the MZR@SPPP formulation measured by high-performance liquid chromatography on day 22 during storage at 4°C (n=2).**

**Figure S8. Ultraviolet-visible absorption spectra of F127 and SPPP.**

**Figure S9. The cell viability of human corneal epithelial (HCE) cells with treatment to different concentrations of MZR (A), SPPP (B), and MZR@SPPP (C) for 24 and 48 hours (n=6). Data are presented as mean ± standard deviation (SD).**

**Figure S10. Representative Live/Dead staining images (A) and quantitative analysis of live cell ratio (B) in HCE cells treated with SPPP and MZR@SPPP (n=3). Data are presented as mean ± SD.**

**Figure S11. The cell viability of HCE cells with treatment to different concentrations of H_2_O_2_ for 3 and 6 hours (n=6). Data are presented as mean ± SD.**

**Figure S12. A volcano plot visualizing the differentially expressed genes (DEGs) between Ctrl and Model groups (A), and between Model and MZR@SPPP groups (B).**

**Figure S13. A Venn diagram showing the overlap of common DEGs between Ctrl vs. Model and Model vs. MZR@SPPP.**

**Figure S14. Gene Ontology (GO) enrichment analysis of DEGs between Ctrl and Model groups (A), and between Model and MZR@SPPP groups (B).**

**Figure S15. Kyoto Encyclopedia of Genes and Genomes (KEGG) pathway enrichment analysis of DEGs between Ctrl and Model groups (A), and between Model and MZR@SPPP groups (B).**

**Figure S16. A heatmap displaying the expression levels of all genes across different groups.**

**Figure S17. Gene set enrichment analysis (GSEA) analysis of the mitogen-activated protein kinase (MAPK) pathway comparing Model and MZR@SPPP groups.**

**Figure S18. The gene expression levels of the pro-inflammatory genes interleukin (IL)-1β (A), IL-6 (B), IL-8 (C), and tumor necrosis factor-α (TNF-α) (D) in HCE cells (n=5). Data are presented as mean ± SD; ***p* < 0.01; *****p* < 0.0001.**

**Figure S19. The western blotting (WB) image (A) and quantitative analysis (B) of p-p38/p38 protein expression (n=5). Data are presented as mean ± SD; ***p* < 0.01; *****p* < 0.0001.**

**Figure S20. A schematic diagram illustrating the process of in vivo safety evaluation.**

**Figure S21. Representative white light and fluorescent staining images (A) and quantitative analysis of corneal fluorescent staining scores (n=6) following twice-daily topical administration for 7 days.**

**Figure S22. Representative images of anterior segment optical coherence tomography (OCT) following twice-daily topical administration for 7 days.**

**Figure S23. Representative images of *in vivo* confocal microscopy following twice-daily topical administration for 7 days.**

**Figure S24. Representative images of fundus photography and posterior segment OCT following twice-daily topical administration for 7 days.**

**Figure S25.** **Representative images of electroretinography (A–C), and quantitative analysis of a- (D) and b- (E) wave amplitudes following twice-daily topical administration for 7 days. Data are presented as mean ± SD.**

**Figure S26. Representative white light and fluorescent staining images (A) and quantitative analysis of corneal fluorescent staining scores (n=6) following twice-daily topical administration for one month.**

**Figure S27. Representative images of anterior segment OCT following twice-daily topical administration for one month.**

**Figure S28. Representative images of *in vivo* confocal microscopy following twice-daily topical administration for one month.**

**Figure S29. Representative images of fundus photography and posterior segment OCT following twice-daily topical administration for one month.**

**Figure S30. Representative images of electroretinography (A–C), and quantitative analysis of a- (D) and b- (E) wave amplitudes following twice-daily topical administration for one month. Data are presented as mean ± SD.**

**Figure S31. Representative hematoxylin and eosin (H&E) staining images of the ocular tissues following twice-daily topical administration for one month.**

**Figure S32. Representative H&E staining images of the liver, kidney, spleen, lung, and heart following twice-daily topical administration for one month.**

**Figure S33. Quantitative analysis of complete blood count parameters following twice-daily topical administration for one month. (A) White blood cell count (WBC). (B) Absolute lymphocyte count (Lymph#). (C) Absolute monocyte count (Mon#). (D) Absolute granulocyte count (Gran#). (E) Red blood cell count (RBC). (F) Hemoglobin concentration (HGB). (G) Hematocrit (HCT). (H) Mean corpuscular volume (MCV). (I) Mean corpuscular hemoglobin (MCH). (J) Mean corpuscular hemoglobin concentration (MCHC). (K) Red cell distribution width (RDW). (L) Platelet count (PLT). (M) Mean platelet volume (MPV). (N) Platelet distribution width (PDW). (O) Plateletcrit (PCT). Data are presented as mean ± SD (n = 3).**

**Figure S34. Quantitative analysis of serum biochemical indicators following twice-daily topical administration for one month. (A) Alanine aminotransferase (ALT). (B) Aspartate aminotransferase (AST). (C) Total bilirubin (TBIL). (D) Albumin (ALB); (E) Alkaline phosphatase (n=3). (F) Creatinine (CREA). Data are presented as mean ± SD (n = 3).**

**Figure S35. *In Vivo* Evaluation of MZR@SPPP Hydrogel Eye Drops in a benzalkonium chloride (BAC)-induced dry eye mouse model. (A) Representative corneal fluorescent staining images following different treatments. (B)** **Quantitative analysis of corneal fluorescent staining scores (n=6). (C) Quantitative analysis of tear production by the Schirmer test (n=6). (D) Quantitative analysis of tear breakup time (TBUT) (n=6). Data are presented as mean ± SD or numbers and percentages (%); **p* < 0.05; ***p* < 0.01; ****p* < 0.001; *****p* < 0.0001.**

**Figure S36. Representative SEM images of the corneal epithelium. Yellow arrow: disrupted organelles. Red arrow: junctional complexes.**

**Supplementary Tables**

**Table S1. Formulations of internal standard solutions**

| **Internal Standard Solution** | **Concentration (μg/mL)** | **Components** |
| --- | --- | --- |
| A | 5000 | 5 mg thiamphenicol + 1 mL methanol |
| B | 500 | 100 μL solution A + 900 μL methanol |
| C | 50 | 100 μL solution B + 900 μL methanol |
| D | 5 | 100 μL solution C + 900 μL methanol |
| E | 0.1 | 1 mL solution D + 49 mL methanol |

**Table S2. Formulations of external standard solutions**

| **External Standard Solution** | **Concentration (μg/mL)** | **Components** |
| --- | --- | --- |
| 1 | 1600 | 5mg MZR + 3.125mL solution E |
| 2 | 160 | 100 μL solution 1 + 900 μL solution E |
| 3 | 16 | 100 μL solution 2 + 900 μL solution E |
| 4 | 1.6 | 100 μL solution 3 + 900 μL solution E |
| 5 | 0.8 | 500 μL solution 4 + 500 μL solution E |
| 6 | 0.4 | 500 μL solution 5 + 500 μL solution E |
| 7 | 0.2 | 500 μL solution 6 + 500 μL solution E |
| 8 | 0.1 | 500 μL solution 7 + 500 μL solution E |
| 9 | 0.05 | 500 μL solution 8 + 500 μL solution E |
| 10 | 0.025 | 500 μL solution 9 + 500 μL solution E |
| 11 | 0.0125 | 500 μL solution 10 + 500 μL solution E |

*Note:* MZR: Mizoribine.

**Table S3. Primers for qRT-PCR.**

| **Gene** | **Sequence** | |
| --- | --- | --- |
| **h-KEAP1** | Forward primer | GTGTCCATTGAGGGTATCCACC |
|  | Reverse primer | GCTCAGCGAAGTTGGCGAT |
| **h-NRF2** | Forward primer | TCAGCGACGGAAAGAGTATGA |
|  | Reverse primer | CCACTGGTTTCTGACTGGATGT |
| **h-SOD2** | Forward primer | GCTCCGGTTTTGGGGTATCTG |
|  | Reverse primer | GCGTTGATGTGAGGTTCCAG |
| **h-HO-1** | Forward primer | AAGACTGCGTTCCTGCTCAAC |
|  | Reverse primer | AAAGCCCTACAGCAACTGTCG |
| **h-IL-1β** | Forward primer | AAGCAGCCATGGCAGAAGTA |
|  | Reverse primer | GGTGGTCGGAGATTCGTAGC |
| **h-IL-6** | Forward primer | CCAGAGCTGTGCAGATGAGT |
|  | Reverse primer | ATTTGTGGTTGGGTCAGGGG |
| **h-IL-8** | Forward primer | GCCAACACAGAAATTATTGTAAAGCTT |
|  | Reverse primer | CCTCTGCACCCAGTTTTCCTT |
| **h-TNF-α** | Forward primer | GTTCCCCAGGGACCTCTCTC |
|  | Reverse primer | GGCTACAGGCTTGTCACTCG |
| **h-β-actin** | Forward primer | ACAGAGCCTCGCCTTTGC |
|  | Reverse primer | GCGGCGATATCATCATCC |
| **m-IL-6** | Forward primer | CCAGAGCTGTGCAGATGAGT |
|  | Reverse primer | ATTTGTGGTTGGGTCAGGGG |
| **m-NOS2** | Forward primer | GCCCAGCCAGCCCAAC |
|  | Reverse primer | GCAGCTTGTCCAGGGATTCT |
| **m-CCL2** | Forward primer | GCCTGCTGTTCACAGTTGC |
|  | Reverse primer | GAGTGGGGCGTTAACTGCAT |
| **m-IL-10** | Forward primer | GCTCCAAGACCAAGGTGTCT |
|  | Reverse primer | AGGACACCATAGCAAAGGGC |
| **m-CHIL3** | Forward primer | AGGGCCCTTATTGAGAGGAG |
|  | Reverse primer | GCACTGTGGAAAAACCGTTGA |
| **m-NRF2** | Forward primer | TCTCCTAGTTCTCCGCTGCT |
|  | Reverse primer | AGTGGGCAACCATCACTCTG |
| **m-HO-1** | Forward primer | CCTCACAGATGGCGTCACTT |
|  | Reverse primer | TGGGGGCCAGTATTGCATTT |
| **m-SOD1** | Forward primer | GTCGGCTTCTCGTCTTGCTC |
|  | Reverse primer | GCTTTCATCGCCATGCTTCC |
| **m-SOD2** | Forward primer | AGGAGAGTTGCTGGAGGCTA |
|  | Reverse primer | TAGTAAGCGTGCTCCCACAC |
| **m-GPX4** | Forward primer | CGCCAAAGTCCTAGGAAACG |
|  | Reverse primer | TATCGGGCATGCAGATCGAC |
| **m-MMP-9** | Forward primer | CGCCACCACAGCCAACTATGAC |
|  | Reverse primer | CCGTCGCTGGTACAGGAAGAGT |
| **m-IL-1β** | Forward primer | GCACTACAGGCTCCGAGATGAA |
|  | Reverse primer | GTCGTTGCTTGGTTCTCCTTGT |
| **m-IFN-γ** | Forward primer | CTTCAGCAACAGCAAGGCGAAA |
|  | Reverse primer | CCGAATCAGCAGCGACTCCT |
| **m-β-actin** | Forward primer | AGATCAAGATCATTGCTCCTCCT |
|  | Reverse primer | ACGCAGCTCAGTAACAGTCC |

**Table S4. Abbreviations.**

| **ABTS** | 2,2′-azinobis(3-ethylbenzothiazoline-6-sulfonic acid) |
| --- | --- |
| **ALB** | Albumin |
| **ALP** | Alkaline phosphatase |
| **ALT** | Alanine aminotransferase |
| **ANOVA** | Analysis of variance |
| **Arg-1** | Arginase-1 |
| **ARVO** | Association for Research in Vision and Ophthalmology |
| **AST** | Aspartate aminotransferase |
| **BAC** | Benzalkonium chloride |
| **BCA** | Bicinchoninic acid |
| **BSA** | Bovine serum albumin |
| **CCK-8** | Cell counting kit-8 |
| **CCL2** | C-C motif chemokine ligand 2 |
| **CD206** | Cluster of differentiation 206 |
| **CD86** | Cluster of differentiation 86 |
| **CDCl_3_** | Deuterated chloroform |
| **CHIL3** | Chitinase-like 3 |
| **CREA** | Creatinine |
| **DAPI** | 4′,6-diamidino-2-phenylindole |
| **CsA** | Cyclosporine A |
| **DBT** | Dibutyltin dilaurate |
| **DCFH-DA** | 2′,7′-dichlorodihydrofluorescein diacetate |
| **DED** | Dry eye disease |
| **DEGs** | Differentially expressed genes |
| **DLS** | Dynamic light scattering |
| **DMEM/F-12** | Dulbecco's Modified Eagle Medium/Nutrient Mixture F-12 |
| **DS** | Desiccating stress |
| **DSC** | Differential scanning calorimetry |
| **EDTA** | Ethylenediaminetetraacetic acid |
| **ELISA** | Enzyme-linked immunosorbent assay |
| **FBS** | Fetal bovine serum |
| **FK506** | Tacrolimus |
| **FRAP** | Ferric reducing antioxidant power |
| **fs** | Femtoseconds |
| **FT-IR** | Fourier transform infrared |
| **GMP** | Guanosine monophosphate |
| **GO** | Gene Ontology |
| **GPC** | Gel permeation chromatography |
| **GPX4** | Glutathione peroxidase 4 |
| **Gran#** | Absolute granulocyte count |
| **GSEA** | Gene Set Enrichment Analysis |
| **HCT** | Hematocrit |
| **H&E** | Hematoxylin and eosin |
| **HGB** | Hemoglobin concentration |
| **H_2_O_2_** | Hydrogen peroxide |
| **HCE** | Human corneal epithelial |
| **HDI** | 1,6-hexamethylene diisocyanate |
| **hEGF** | Human epidermal growth factor |
| **HO-1** | Heme oxygenase-1 |
| **HPLC** | High-performance liquid chromatography |
| **HPLC-MS/MS** | High-performance liquid chromatography-mass spectrometry/mass spectrometry |
| **IFN-γ** | Interferon-gamma |
| **IL-10** | Interleukin-10 |
| **IL-13** | Interleukin-13 |
| **IL-17** | Interleukin-17 |
| **IL-1β** | Interleukin-1beta |
| **IL-4** | Interleukin-4 |
| **IL-6** | Interleukin-6 |
| **IL-8** | Interleukin-8 |
| **IMPDH** | Inosine monophosphate dehydrogenase |
| **iNOS** | Inducible nitric oxide synthase |
| **K_D_** | Kinetic constant |
| **KEAP1** | Kelch-like ECH-associated protein 1 |
| **KEGG** | Kyoto Encyclopedia of Genes and Genomes |
| **KRT10** | Keratin 10 |
| **KRT12** | Keratin 12 |
| **LPS** | Lipopolysaccharide |
| **Lymph#** | Absolute lymphocyte count |
| **MAPK** | Mitogen-activated protein kinase |
| **MCH** | Mean corpuscular hemoglobin |
| **MCHC** | Mean corpuscular hemoglobin concentration |
| **MCV** | Mean corpuscular volume |
| **MMP** | Mitochondrial membrane potential |
| **MMP-3** | Matrix metalloproteinase-3 |
| **MMP-9** | Matrix metalloproteinase-9 |
| **Mon#** | Absolute monocyte count |
| **MPV** | Mean platelet volume |
| **MUC1** | Mucin-1 |
| **MZR** | Mizoribine |
| **NF-κB** | Nuclear factor kappa-B |
| **NMR** | Nuclear magnetic resonance |
| **NRF2** | Nuclear factor erythroid 2-related factor 2 |
| **OCT** | Optical Coherence Tomography |
| **ns** | Nanoseconds |
| **OD** | Optical density |
| **PAS** | Periodic acid–Schiff |
| **PBS** | Phosphate buffered saline |
| **PCT** | Plateletcrit |
| **PDMS** | Polydimethylsiloxane |
| **PDW** | Platelet distribution width |
| **PEG** | Polyethylene glycol |
| **PEO** | Polyethylene oxide |
| **PLT** | Platelet count |
| **PPG** | Polypropylene glycol |
| **PPO** | Propylene oxide |
| **PVDF** | Polyvinylidene difluoride |
| **qPCR** | Quantitative polymerase chain reaction |
| **qRT-PCR** | Quantitative real-time polymerase chain reaction |
| **RBC** | Red blood cell count |
| **RDW** | Red cell distribution width |
| **Rg** | Radius of gyration |
| **Rho** | Rhodamine |
| **RIPA** | Radioimmunoprecipitation assay |
| **RMSD** | Root mean square deviation |
| **RMSF** | Root mean square fluctuation |
| **RNA-seq** | RNA sequencing |
| **ROS** | Reactive oxygen species |
| **SASA** | Solvent accessible surface area |
| **SD** | Standard deviation |
| **Se** | Selenium |
| **SEM** | Scanning electron microscope |
| **SOD** | Superoxide dismutase |
| **SOD1** | Superoxide dismutase 1 |
| **SOD2** | Superoxide dismutase 2 |
| **SPP** | poly(Se-PEG-PPG) |
| **SPPP** | Poly (PDMS-SE-PEG-PPG) |
| **SPR** | Surface plasmon resonance |
| **TBIL** | Total bilirubin |
| **TBUT** | Tear breakup time |
| **Tg** | Glass transition temperature |
| **TGA** | Thermogravimetric analysis |
| **TIP3P** | Transferable Intermolecular Potential 3-Point |
| **TNF** | Tumor necrosis factor |
| **TNF-α** | Tumor necrosis factor-alpha |
| **TOMM20** | Translocase of outer mitochondrial membrane 20 homolog |
| **TUNEL** | Terminal deoxynucleotidyl transferase–mediated deoxyuridine triphosphate nick-end labeling |
| **WB** | Western blotting |
| **WBC** | White blood cell count |
